# *In silico* Prediction of Skin Sensitization: *Quo vadis*?

**DOI:** 10.3389/fphar.2021.655771

**Published:** 2021-05-04

**Authors:** Giang Huong Ta, Ching-Feng Weng, Max K. Leong

**Affiliations:** ^1^ Department of Chemistry, National Dong Hwa University, Shoufeng, Taiwan; ^2^ Department of Basic Medical Science, Institute of Respiratory Disease, Xiamen Medical College, Xiamen, China

**Keywords:** skin sensitization, in silico models, human test methods, non-animal test methods, animal test methods, in chemico test methods

## Abstract

Skin direct contact with chemical or physical substances is predisposed to allergic contact dermatitis (ACD), producing various allergic reactions, namely rash, blister, or itchy, in the contacted skin area. ACD can be triggered by various extremely complicated adverse outcome pathways (AOPs) remains to be causal for biosafety warrant. As such, commercial products such as ointments or cosmetics can fulfill the topically safe requirements in animal and non-animal models including allergy. Europe, nevertheless, has banned animal tests for the safety evaluations of cosmetic ingredients since 2013, followed by other countries. A variety of non-animal *in vitro* tests addressing different key events of the AOP, the direct peptide reactivity assay (DPRA), KeratinoSens™, LuSens and human cell line activation test h-CLAT and U-SENS™ have been developed and were adopted in OECD test guideline to identify the skin sensitizers. Other methods, such as the SENS-IS are not yet fully validated and regulatorily accepted. A broad spectrum of *in silico* models, alternatively, to predict skin sensitization have emerged based on various animal and non-animal data using assorted modeling schemes. In this article, we extensively summarize a number of skin sensitization predictive models that can be used in the biopharmaceutics and cosmeceuticals industries as well as their future perspectives, and the underlined challenges are also discussed.

## Introduction

Skin is a protective barrier against the external environment to guard internal organs, bones, and muscles. The skin is an organ of the integumentary system made of multiple layers containing *epidermis* (surface layer), and dermis (deeper layer) (Rehfeld et al., 2017). The topical application and transepidermal delivery of natural or synthesized chemicals are striking approaches for the discovery and development of drugs and medicines by physicians and pharmacologists (Alkilani et al., 2015); and for maintaining healthy skin in general by dermatologists (Kraft and Lynde, 2005; Spada et al., 2018). Upon skin contacts with chemicals or substances that could be non-allergy or allergen caused the hypersensitivity to the subject (Lee and Thomson, 1999). There are four types of skin hypersensitivity, fundamentally, based on the immunologic mechanism that mediates the disease, namely type I (immediate/IgE-related), in which the cutaneous skin test reaction reaches the peak at 2 h; type II (antibody and complement related cytotoxicity); type III (antigen-antibody complex mediated); and type IV or delayed type hypersensitivity (DTH) response, that can occur within 48–72 h (Lee and Thomson, 1999; Posadas and Pichler, 2007). Notably, skin sensitization or allergic contact dermatitis (ACD) is a type IV DTH or type IV allergy (Ouyang et al., 2014). ACD can substantially affect the life quality of patients with uncomfortable symptoms of skin rash, blister, and/or swollen that could persist for a lifetime in some cases (Strickland et al., 2016). In illness observation, ACD has affected more than 20% of North America’s and Western Europe’s population based on the data collected from all age groups, and the contact allergy tends to be more prevalent in younger children as the comparison with adults (Thyssen et al., 2007).

The adverse outcome pathways (AOPs) of skin sensitization are the sequential events from the initial skin exposure to chemicals, followed by triggering the downstream cascade pathways, which include induction and elicitation phases. The chemical sensitization pathway (CSP) is initialized by the adduct formation, *viz*. a covalent bond between skin proteins and chemicals to subsequently form a full antigen (Enoch et al., 2011). Moreover, skin sensitizers have the same physicochemical properties as haptens, which are electrophilic *per se* (Roberts and Lepoittevin, 1998) or lead to the formation of free radicals (Gäfvert et al., 1994). In some cases, skin sensitizers can be named as prehapten, which initially are not electrophilic or radicals but can be activated through air exposure, photoactivation, bacterial degradation on the skin surface (Karlberg et al., 1992; Sköld et al., 2002) and skin sensitizers also can be termed as prohapten, which can be triggered through the metabolic pathway (Nilsson et al., 2005; Gerberick et al., 2008; van Eijl et al., 2012). As such, those skin sensitizers may act as electrophiles, whereas the skin protein functions as a nucleophile in the process of adduction formation. More specifically, those nucleophilic amino acids such as cysteine (thiols), histidine, lysine (primary amines), methionine, and tyrosine within the skin protein can interact or react with electrophilic hapten (Ahlfors et al., 2003; Gerberick et al., 2004; Schwöbel et al., 2011). This interaction with cysteine and/or lysine leads to the formation of covalent bonds and production of the hapten‒protein complex consequently processed by both epidermal and dermal dendritic cells (DCs), which constitute the skin immune system (Ochoa et al., 2008; Clausen and Stoitzner, 2015). Subsequently, the DC presents the part of this protein complex (antigen) on major histocompatibility complex (MHC) and activates naïve T lymphocyte in the lymph node (Martin et al., 2010; Huppert et al., 2018; Johnson et al., 2020). In addition, this can induce the differentiation and proliferation of T cells that, in turn, will propagate the inflammatory response throughout the whole body (Gefen et al., 2015). After the initial exposure, the secondary exposure to the same allergen will initiate the elicitation phase, in which the activated T cells are triggered to secrete specific cytokines to attract inflammatory cells entering into the *epidermis* of infected parts, causing rash, itchy, and burning on the exposed skin surface. The detail of induction and elicitation phases has been illustrated elsewhere (OECD, 2012). Markedly, the response in the elicitation phase of the immune system is faster than that in the induction phase (OECD, 2012).

The complications of whiting-cosmetics have been documented in recent years since the cosmetic ingredients are not only a major concern in the beauty industry but also a critical factor in human health. Some ingredients in brightening cosmetics such as hydroquinone, corticosteroids, and mercury can cause severe complications. For instance, chronic application of hydroquinone-contained cosmetics can result in exogenous ochronosis or “fish odor syndrome,” the accumulation of mercury can lead to increased pigmentation, nail discoloration, and ACD; and the aggregation of corticosteroids will produce “steroid addiction syndrome” or induce acne on the anterior chest (Olumide et al., 2008; Ladizinski et al., 2011; Mahé, 2014). Cosmeceuticals is a burgeoning industry, in which cosmetic products can exert therapeutic effects (Martin and Glaser, 2011). Vitamin, hydroxy acids, growth factors, peptides, and botanicals, for example, are considered as the cosmeceutical ingredients (Martin and Glaser, 2011). Some skincare products also include ingredients with pharmaceutical properties as exemplified by Oz.Or. Oil 30, which cannot only soften the skin but also show the potential in antibacteria and ameliorating dermal wound healing (Serio et al., 2017). Sargafuran, which is extracted from marine brown alga, is a promising compound to be used in skincare cosmetics for preventing acne because of its antibacterial properties (Kamei et al., 2009). In addition, Food and Drug Administration (FDA) has already approved some antibiotics such as quinupristin-dalfopristin, linezolid, and daptomycin for the treatment of skin-structure infections (Schweiger and Weinberg, 2004). According to FDA regulation, a product can be both a cosmetic and a drug if it can meet the definitions of both cosmetics and drugs. However, the category such as “cosmeceuticals” has not been recognized by FDA. In contrast, cosmeceuticals are a subclass of cosmetics in Europe and Japan, and considered as a subclass of drugs in the UK (Pandey et al., 2021). However, the criteria for classifying compounds or raw materials from the same plant are various in different countries. For instance, the raw materials of *C. limon* have been considered as the natural ingredients to potentially threat human health by the European Food Safety Authority (EFSA). However, the oil and extracts from this species is classified as safe products by FDA (Klimek-Szczykutowicz et al., 2020), suggesting that these criteria are not universally applicable.

**FIGURE 1:**
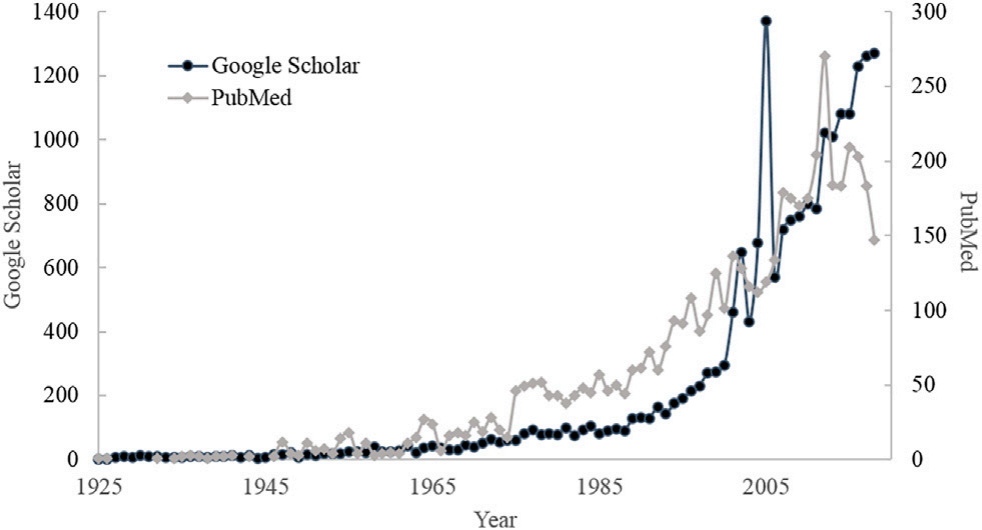
The number of publications searched by Google Scholar and PubMed with the keyword “Skin sensitization.”

Skin sensitization is an increasingly important issue that can be manifested by the number of publications about skin sensitization as illustrated in Figure 1. It can be observed that the number of published literature has gradually increased in recent years, especially the dramatic increase after 2000. The consumption and interest in the cosmetic market have progressively increased by 5% every year and it is expected to reach 31.75 billion US dollars by 2023 (Kumar, 2005; Orbis-Research, 2018). The potential benefits and demand are still high and the information about toxicity, physicochemical, and bioactivity properties of the cosmetics ingredients need to be promoted (Kumar, 2005; Panico et al., 2019), the growth of the global cosmetics market is updated annually at http://www.statista.com/statistics/297070/growth-rate-of-the-global-cosmetics-market/.

## Skin Sensitization Assay

Various tests have been devised to evaluate the potential of the human skin sensitization of new substances and they can be basically classified into human tests, animal tests, and non-animal tests as enlisted in Table 1. The more detailed test information will be discussed as follows.

**TABLE 1:** Animal and non-animal tests to evaluate the potential of the human skin sensitization of new substance depending on key events in AOP (OECD, 2012).

Key event	Function and focus	Method	References
1	The molecular interaction with skin proteins through cysteine and/or lysine residue	DPRA, kDPRA, ADRA	OECD (2015a), OECD (2020)
2	The inflammatory response through keratinocyte	KeratinoSens™	OECD (2017), OECD (2018a)
3	The activation of dendritic cells	h-CLAT	OECD (2016b), OECD (2018c)
		U-SENS^TM^	
4	The proliferation of T cells	LLNA	OECD (2010a), OECD (2010b)

OECD: Organization for Economic Co-operation and Development (France, 1961).

### Human Tests

Human tests for skin sensitization include human repeat insult patch test (HRIPT) and human maximization test (HMT) (Kligman, 1966; Kligman and Epstein, 1975; Marzulli and Maibach, 1974). In both tests, the human skin reaction is recorded after the secondary contact between a tested substance and human skin. Generally, the response of the tested substance is classified into 5 levels according to the incidence of the positive response from test subjects in the HMT system: weak (0–2/25), mild (3–7/25), moderate (8–13/25), strong (14–20/25), or extreme sensitizer (21–25/25) (Kligman, 1966). The HRIPT classification system is instituted according to the grades of skin reactions: 1) erythema; 2) erythema and induration; 3) vesiculation; and 4) bulla formation and only the substances of grade 1 are qualified as non-sensitizers (Marzulli and Maibach, 1974). To date, the classification systems of skin sensitization in human tests are not consistent and often depend on the subjective judgment of experts (Gerberick et al., 2001; Roberts, 2018). In the classification and labeling of chemicals of the globally harmonized system (GHS), chemicals are classified as subcategory 1A or 1B if their HRIPT or HMT values are ≤500 μg/cm^2^ or HRIPT or HMT values are >500 μg/cm^2^, respectively. Both subcategories are experimentally considered as skin sensitizers. Non sensitizers are not classified in this classification system (ICCVAM, 2011; United Nation, 2013). Nevertheless, the other classification system has also been proposed, in which chemicals are classified into six skin sensitization categories based on their no observed effect level (NOEL) values of HRIPT as enlisted in Table 2 (Basketter et al., 2014). Nowadays, human tests are only implemented to confirm skin sensitization of test chemicals under specific conditions and no maximum concentrations are allowed to apply due to ethical issues (Kimber et al., 2001).

**TABLE 2:** The skin sensitization categories based on the NOEL value of HRIPT.

Category	Characteristics	HRIPT NOEL value
1	High intrinsic skin sensitization potency	Less than 25 μg/cm^2^
2	Less sensitizing than category 1, the contact with moderate concentration can trigger 1–10% positive induction of subjects	Between 25 and 500 μg/cm^2^
3	Substances known as contact allergens produce sensitization in 0.01–0.1% of those exposed	Between 500 and 2,500 μg/cm^2^
4	Chemicals in this category require prolonged exposure to higher dose level to produce sensitization and are rarely regarded as important clinical allergens	More than 2,500 μg/cm^2^
5	Very low intrinsic ability to cause skin sensitization. Even in the highly selected patient groups, the incidence should not exceed 1%	The NOEL values are variable or absents, because of the inaccuracy of determination of a threshold
6	Free from skin sensitization activity	

### Animal Tests

Besides human tests, there are various animal tests have been conducted to evaluate the potential of the human skin sensitization for new substance, namely local lymph node assay (LLNA), which depends on the nature of AOP key events as listed in Table 1 (OECD, 2012), Guinea pig maximization test (GPMT), and Buehler tests. Of various animal assay systems, LLNA (OECD, 2010a; 2010b), which is based on the extent of induced proliferative responses in draining lymph nodes after the topical exposure of chemicals to mice (stimulation index, SI), is the preferred animal test model and has been adopted by various regulatory agencies (Cockshott et al., 2006; Gerberick et al., 2007a). The LLNA system is designated to measure the substance concentration when the lymphocyte proliferation of the lymph node is three-fold higher than that of the vehicle-treated controls, *viz*. SI ≧ 3, and is defined as the LLNA EC3 value. The risk potential of skin sensitizers is categorized into various classes according to the measured LLNA EC3 values as summarized in Table 3 (ICCVAM, 2011). The LLNA EC3 value has been converted from the percentage to µg/cm^2^ since 2001 to develop the correlation between LLNA EC3 and NOEL value from human tests, namely HRIPT and HMT. It has been found that the EC3 value can be used to quantitatively estimate the skin sensitization potency in human since EC3 values can be highly correlated with NOELs (Gerberick et al., 2001) that also has been confirmed by Api et al. (Api et al., 2015).

**TABLE 3:** The skin sensitization potency based on LLNA EC3 values.

Potency category	Threshold (%)
Extreme	EC3 < 0.1
Strong	0.1 ≤ EC3 <1
Moderate	1 ≤ EC3 <10
Weak	10 ≤ EC3 ≤100

The GPMT method is another popular animal model, in which intradermal injection and/or epidermal application were employed in induction periods to expose guinea pig skin with the test substances. The animals are repeatedly exposed to test substances with a challenge dose after 10–14 days. The skin reaction to the challenge exposure in the test animals is determined by comparing it with the untreated control animals (OECD, 1992). The skin sensitization potential of chemicals can be classified into various extents, namely extreme, strong, moderate, or weak levels, depending on the induction concentration and the incidence of subjects as listed in Table 4 (ICCVAM, 2011). The Buehler method is another test to use guinea pig skin as a module. The only difference between GPMT and Buehler test is the way of sample preparation in that the test substance is mixed with Freund’s complete adjuvant (FCA) in the GPMT test, whereas that step is absent in the non-adjuvant Buehler method (OECD, 1992). Moreover, LLNA and GPMT can be carried out in a synergistic fashion to evaluate skin sensitization. More specifically, there is no need to carry out the GPMT or Buehler test for further validation once the test substance is defined as skin sensitization positive by LLNA. Nevertheless, a substance is subjected to further evaluation by GPMT or Buehler test in case it is qualified as skin sensitization negative by LLNA (OECD, 1992).

**TABLE 4:** The skin sensitization potential based on GPMT.

Induction concentration (%)	GPMT incidence (%)	
	30 to <60	≥60
<0.1	Strong	Extreme
≥0.1 to <1	Moderate	Strong
≥1 to <10	Weak	Moderate
≥10 to ≤100	Weak	Weak

The LLNA and GPMT skin sensitization models cannot completely serve as a surrogate to predict skin sensitization potential in human since they can only accurately predict 70% of human tests in addition to the fact that LLNA and GMPT do not always reach the same agreement. It has been shown that the LLNA model can foretell human skin sensitization better than GPMT in case of discordance between LLNA and GPMT assays (Dean et al., 2001). As such, animal tests cannot completely replace their human counterparts because of their limitations. Surprisingly, it has been observed that one-third of strong sensitizers in the human test were predicted to be weak sensitizers by LLNA despite the fact that the LLNA test is commonly recognized as the gold standard for the human skin sensitization test (ICCVAM, 2011; Strickland et al., 2017; Roberts and Api, 2018), indicating the discrepancy in both assay systems. In addition, LLNA predictions can correlate with human tests well as long as those sensitizers lie within the applicability domain of the LLNA model. The inconsistent predictions, nevertheless, are not due to randomness. More specifically, the under-estimation of human skin sensitization potency by the LLNA model can be principally attributed to the fact that the test chemicals contain electrophilic aromatic Schiff bases or impurities (Roberts and Api, 2018). Conversely, the LLNA model is prone to over-estimating the potency when compared with the human test if the test chemicals under LLNA conditions will undergo autoxidation or have cutaneous pharmacological potentials other than skin sensitization (Roberts and Api, 2018). Furthermore, chemicals, namely pre- or pro-haptens, can be falsely predicted (Roberts and Api, 2018). Moreover, animal tests for skin sensitization that have been adopted for a long time still comprised some controversial issues concerning their effectiveness and ethical problems (Rollin, 2003). In 2017, Predictive Toxicology Roadmap was established by FDA. In this project, they evaluate new methods and technologies that can expand the predictive capabilities of toxicology and reduce the use of animal testing. With the same goals, the *in vitro* testing methods have been evaluated by a Consortium comprising the Institute for *In vitro* Sciences, Inc (IIVS) the Consumer Healthcare Products Association (CHPA), and the PETA International Science Consortium (PETA-ISC) to substitute rabbit vaginal irritation (RVI) test (Costin et al., 2020). There is a growing tendency, nevertheless, to use non-animal tests as an alternative approach to assess skin sensitization (Doke and Dhawale, 2015).

### Non-animal Assays

Animal testing approaches for cosmetic products have been banned due to animal rights and welfare by the 7th amendment to the EU Cosmetics Directive in Europe since 2013 (European Union, 2003; European Commission, 2013a). Notably, some non-animal testing methods have been developed to resolve this challenge and approved by the European Union Reference Laboratory for alternatives to animal testing (EURL ECVAM) (European Commission, 2013a; European Commission, 2013b). Nonetheless, non-animal test data may still have limitations in predicting skin sensitization indeed. For instance, those methods accepted by the Organization for Economic Co-operation and Development (OECD) only focus on one AOP key event or activation of some specific genes, such depletion of cysteine and/or lysine-containing protein in Direct Peptide Reactivity Assay (DPRA) (OECD, 2015a), CD86 and CD54 overexpression in human cell line activation test (h-CLAT) (OECD, 2018c), induction of nuclear factor-erythroid-2 related factor 2 (Nrf2)-Kelch-like ECH-associated protein 1 (Keap1)-antioxidant/electrophile response element (ARE) pathway in KeratinoSens™ (OECD, 2015b), CD86 overexpression for U-SENS™ test (OECD, 2016b), and the expressions of anti-oxidation, inflammation, and cell migration genes in SENS-IS test (Cottrez et al., 2016).

DPRA is a non-animal model focused on the hapten and protein interaction due to skin exposure to chemical substances. This method was first proposed by Gerberick and has been further accepted by OECD since 2015 (Gerberick et al., 2004; Gerberick et al., 2007b; OECD, 2015a), in which the synthesized peptides such as cysteine (Ac-RFAACAA-COOH) and lysine (Ac-RFAAKAA-COOH) (R: Arginine, F: Phenylalanine, A: Alanine, C: Cysteine K: Lysine) are incubated with test substances, followed by measuring the absorption peaks at 220 nm to determine the concentration of cysteine and lysine after the reaction. Generally, skin sensitization can be divided into 4 classes, namely negative, positive with low, moderate, or high reactivity level (OECD, 2015a). Nevertheless, DPRA can be limited by solubility and complex mixture (Gerberick, 2016). In addition, the accuracy of measurement results would be hampered by the fact that chemicals could be co-eluted with the peptide (Natsch et al., 2007; Natsch and Gfeller, 2008). Until now, the DPRA has been improved by another version such as amino acid derivative reactivity assay (ADRA) to prevent the co-elution of test chemicals and nucleophilic agents (Fujita et al., 2019; Wanibuchi et al., 2019; Imamura et al., 2021). This method has been accepted by OECD (OECD, 2020). Another modified DPRA called kinetic direct peptide reactivity assay (kDPRA), in which several concentrations of tested compounds are incubated with synthetic peptide for different incubation times, has been accepted by OECD (OECD, 2020). The matrix of depletion values and incubation times and concentrations are constructed to measure the rate constant (log *k*_max_). The test compounds are further classified into GHS classification scales by their log *k*_max_ values. (Natsch et al., 2020). The reproducibility between intra- and inter-laboratories for this method achieved 96 and 88%, respectively (Wareing et al., 2020).

The KeratinoSens™ method takes a different approach by focusing on a second AOP key event (Table 1), namely the inflammatory responses and gene expression associated with specific cell signaling pathways such as ARE-dependent pathways. Keap1 binds to the transcription factor Nrf2 in the un-induced state that helps ubiquitin bind to Nrf2 by CuI2-mediated ubiquitinylation, which, in turn, can degrade Nrf2 into the proteasome (de Freitas Silva et al., 2018). However, Keap1 cannot bind to Nrf2 protein once the covalent bond is formed between Keap1 and small molecules such as sensitizers, leading to the accumulation of Nrf2 protein in the nucleus. The released Nrf2 protein binds to ARE sequence in the promoter regions of detoxification, antioxidant, and anti-inflammatory genes, triggering the expression of target genes (de Freitas Silva et al., 2018). The mechanism of Nrf2-ARE pathway activation was illustrated in Figure 2 of de Fritas Silva et al. (de Freitas Silva et al., 2018). Accordingly, the human keratinocytes HaCaT cells are stably transfected with the selected plasmid, which contains the ARE sequence, SV40 promoter, and luciferase gene (*luc2*) in the KeratinoSens™ test (Emter et al., 2010; Steinberg, 2013). The test chemicals are designated as sensitizers in the KeratinoSens™ test provided that they can produce the induction of luciferase activity above 1.5 folds with respect to the negative control or non-sensitizers otherwise (OECD, 2015b). The LuSens assay is another method accepted by OECD and is developed based on the same concept as KeratinoSens™ (Ramirez et al., 2014; OECD, 2018b).

The activation of DCs is the AOP key event investigated in the h-CLAT method (Table 1). In the induction phase of skin sensitization, the co-expression of CD86 and CD54 on the Langerhans cells is used as the indicator of the antigen-presenting process (Nuriya et al., 1996; Reiser and Schneeberger, 1996; Tuschl et al., 2000). Thus, the expressions of CD86 and CD54 on THP-1 cells, which are a human monocytic leukemia cell line, are measured in the event when THP-1 cells are exposed to sensitizers in the h-CLAT method (Sakaguchi et al., 2006; Sakaguchi et al., 2009). The upregulation of these markers indicates the occurrence of DCs activation and the skin sensitization activity caused by the test chemical. Of note, chemicals are further classified as sensitizers or non-sensitizers in this test (OECD, 2018c). In addition, the U-SENS™ test method for skin sensitization testing is based on the expression of CD86 cell surface marker on the U937 cells, which are a human histiocytic lymphoma cell line (Piroird et al., 2015). Briefly, a compound is considered to be a sensitizer when CD86 expression in the U937 cell line is 1.5 fold higher than the untreated control and non-sensitizer otherwise. It is of interest to note that this method has been submitted to OECD and the drafted proposal has been publicized on the OCED website (OECD, 2016b; OECD, 2018c). This method recently has been used to evaluate the role of nanomaterials in skin sensitization (Bezerra et al., 2021).

SENS-IS is another non-animal method to measure the skin sensitivity of chemicals using the commercially reconstituted human skin (EpiSkin) (Netzlaff et al., 2005; Cottrez et al., 2015), in which the expression levels of Redox and SENS-IS genes are measured. The former includes 17 genes that contained an ARE in their promoter (Cottrez et al., 2016), which are related to the target genes modulated by the Nrf2-Keap1-ARE signaling pathway, whereas the latter includes 21 genes, which are linked to the activities of DCs and associated with inflammation, danger signals, and cell migration. Those genes measured in the SENS-IS group can be triggered by sensitizers but not under the control of the Nrf2-Keap1-ARE pathway (Cottrez et al., 2015; Cottrez et al., 2016). There are four chemical concentrations, namely 50, 10, 1, and 0.1%, applied onto the artificial skin, followed by collecting the mRNA from the EpiSkin cells and analyzing the gene expression using reverse transcriptase polymerase chain reaction (RT-PCR). When the number of expressed genes is more than 7 and less than 20 in both groups, the test chemicals are defined as sensitizers and subsequently categorized as weak, moderate, strong, or extreme sensitivity depending on the chemical concentrations that, in fact, is similar to the classification system adopted by LLNA. The test chemical concentration will be lowered when there are 20 genes expressed that are termed overexpression. Moreover, a chemical is considered as negative in case of failures in all tested concentrations (Cottrez et al., 2016). Until now, this method is still under the validation process, which has been announced at https://tsar.jrc.ec.europa.eu/.

Various research groups have published their assay data using those above-mentioned methods and the results are summarized in Table 5, which provides affluence of data source for building *in silico* models. Until now, there are still many researchers endeavoring to improve the accuracy of *in vitro* assay for assessing the skin sensitization potential such as finding new biomarkers for predicting skin sensitization (Hirota and Moro, 2006) or developing a novel assay like Genomic Allergen Rapid Detection (GARD™) to define the skin sensitization activity by only one assay (Roberts, 2018). GARD™ depends on the changes of the gene expression when myeloid cells are exposed to the chemicals (Johansson et al., 2013; Johansson et al., 2019; Johansson et al., 2011; Roberts, 2018; Stevenson et al., 2019). This method was validated by numerous laboratories with an inter-laboratory reproducibility of 92.0% in 2019 (Johansson et al., 2019), and has been under the peer-review process for EURL ECVAM validation, which has been announced at https://tsar.jrc.ec.europa.eu/. Furthermore, the conformal prediction has been implemented into GARD™ protocol with an accuracy of 88%. In 2021, Masinja et al., nevertheless, used GARD™ to predict the skin sensitization potential of agrochemical active ingredients in total 7/12 GARD™ results concurred with mammalian data, suggesting that GARD™ still needs to be improved to validate the skin sensitization potential of not only cosmetics, but also the other active ingredients (Masinja et al., 2021). Another way is to modify or to improve the current non-animal methods to further increase the accuracy. For instance, the spectro-DPRA method using 5, 5-dithiobis-2-nitrobenzoic acid, or fluorescamine™ as the detection reagent was designed to investigate the unreacted peptide in 2014. It was demonstrated that the accuracy of this method could increase up to 91.5 and 94.9% when compared with LLNA and human data, respectively (Cho et al., 2014; Cho et al., 2019).

**TABLE 5:** Non-animal skin sensitization assay types and data sources.

Assay type	Data sources
DPRA	Bauch et al. (2011), Bauch et al. (2012), Gerberick et al. (2004), Gerberick et al. (2007b), Hoffmann et al. (2018), Jaworska et al. (2013), Jaworska et al. (2011), Natsch et al. (2013), Nukada et al. (2013), Takenouchi et al. (2015), Urbisch et al. (2015)
h-CLAT	Ashikaga et al. (2010), Sakaguchi et al. (2010), Bauch et al. (2011), Nukada et al. (2011), Bauch et al. (2012), Nukada et al. (2013), Takenouchi et al. (2013), Takenouchi et al. (2015), Urbisch et al. (2015), Hoffmann et al. (2018)
KeratinoSens™	Emter et al. (2010), Ball et al. (2011), Bauch et al. (2011), Bauch et al. (2012), Jaworska et al. (2013), Natsch et al. (2013), Urbisch et al. (2015), Hoffmann et al. (2018)
SENS-IS	Cottrez et al. (2016), Hoffmann et al. (2018)
U-SENS^TM^	Python et al. (2007), Bauch et al. (2011), Bauch et al. (2012), Jaworska et al. (2013), Natsch et al. (2013), Piroird et al. (2015), Hoffmann et al. (2018)

Most of the non-animal tests such as U-SENS™, h-CLAT, and KeratinoSens™ are qualitative *per se*, in which compounds are divided into skin sensitization positive and negative, *viz*. a binary classification fashion (OECD, 2015b; 2016b; 2018c), whereas DPRA and SENS-IS are basically quantitative, in which the levels of skin sensitization potential are determined (OECD, 2015a). Additionally, the non-animal tests such as DPRA, h-CLAT, and KeratinoSen™ are routinely used as the preliminary screening by Europe, whereas others such as U-SENS™ and SENS-IS can be implemented to further characterize the nature of skin sensitization (OECD, 2016b, 2019). The non-animal models for skin sensitization have been adopted for a long time and the first non-animal DPRA model has been accepted by OECD since 2015. However, not all chemicals such as insoluble chemicals, pro-haptens, and chemicals co-eluting with the model peptide can be assessed by DPRA that may severely limit their applications. These chemicals, nevertheless, can be evaluated by *in silico* models in the preliminary phase (Urbisch et al., 2016). As such, *in silico* models are expected to be a useful method for predicting skin sensitization of novel chemicals in this aspect.

## *In silico* Models

### Data Source

Europe has banned animal tests to verify the safety of cosmetic products such as toxicity in repeated dose systems, skin sensitization, reproductive toxicity, carcinogenicity, and toxicokinetics since 2013 (European Commission, 2013a). Alternatively, various non-animal tests, namely DPRA, KeratinoSens™, and h-CLAT, have been derived and accepted by OCED (*vide supra*). In addition, various skin sensitization data have been published according to the collection of animal and non-animal data, as well as chemical structure information are listed in Table 5. Some online skin sensitization data sources, which have collected the data and structural alerts, can be used to build predictive models and are listed in Table 6. Of various skin sensitization databases, *SkinSensDB*, which has collected the animal and non-animal tests and contains 710 unique chemicals with 2,078, 467, 1,323, and 1,060 assay values for peptide reactivity (DPRA), keratinocyte activation (KeratinoSen™), dendritic cell activation (h-CLAT), and T-cell activation (LLNA-EC3), respectively (Tung et al., 2019; Wang et al., 2017), is freely accessible. The non-confidential substance data, which have been submitted to European chemicals agency (ECHA) under the Registration, Evaluation, Authorization and Restriction of Chemicals (REACH) regulation, are publicly available and free of charge. This dataset contains many types of assays and study categories. Another available source is Cosmetic ingredient database (CosIng). This European Commission database includes the information about cosmetic substances and ingredients (Table 6). Another source is Chemical Evaluation and Risk Estimation System (CERES), which is developed by FDA’s Center for Food Safety and Nutrition. This database contains toxicity data including the skin sensitization hazard and potency (Ghosh et al., 2020).Various predictive models and packages to predict skin sensitization have been published (Wilm et al., 2018). Some models provide structure alerts based on the analysis of chemical characteristics that are responsible for skin sensitization (Sushko et al., 2011). *ToxAlerts* was established in 2012 serving as a valuable data source for model development to predict the chemical toxicity. Initially, 600 structural alerts for carcinogenicity, mutagenicity, skin sensitization, acute aquatic toxicity, and potential idiosyncratic drug toxicity were issued (Sushko et al., 2012), and the number has increased to more than 3,000 structural alerts to date. The Interagency Coordinating Committee on the Validation of Alternative Methods (ICCVAM) is a permanent committee of the National Toxicology Program (NTP) Division, which is responsible for evaluating the toxic potential, developing and validating the toxicology methods, collecting the data to strengthen the scientific base for risk assessment. ICCVAM has established a database for skin sensitization with the collection of 1,060 chemicals for the LLNA test and 208 chemicals for the GPMT and Buehler tests (ICCVAM, 2011). Vitic is a commercial toxicity database and information management system developed by Lhasa, consisting of more than 38,000 skin sensitization data for more than 10,000 structures. eChemPortal, which has been developed by OECD, is a free public source and provides the chemical characteristics of physical-chemical properties, environmental fate and behavior, ecotoxicity, and toxicity. The chemical information can be searched using chemical names and numbers or GHS classifications.

**TABLE 6:** Online skin sensitization databases.

database	Website
*SkinSensDB*	http://cwtung.kmu.edu.tw/skinsensdb
*Toxalert*	http://ochem.eu/alerts
National Toxicology Program	https://ntp.niehs.nih.gov/whatwestudy/niceatm/test-method-evaluations/immunotoxicity/llna/index.htmlhttps://ntp.niehs.nih.gov/iccvam/methods/immunotox/niceatm-llnadatabase-23dec2013.xls
Vitic	https://www.lhasalimited.org/products/vitic.htm
	https://www.lhasalimited.org/products/skin-sensitization-assessment-using-derek-nexus.htm
eChemPortal	https://www.echemportal.org/echemportal/index.action
REACH	https://iuclid6.echa.europa.eu/reach-study-results
CosIng	https://ec.europa.eu/growth/sectors/cosmetics/cosing_en
CERES	https://www.accessdata.fda.gov/scripts/fdatrack/view/track_project.cfm?program¼cfsan&id¼CFSANOFAS-Chemical-Evaluation-and-Risk-Estimation-System

### Commercial Package

Numerous commercially available packages and/or models have been published and are listed in Table 7. Computer automated structure evaluation (case) Ultra program is a commercial package to issue the structure alerts, in which, principally, molecular structures are divided into various subunits and those ones responsible for specific activities are identified and termed biophore (Klopman, 1992). Moreover, different subunits can give rise to different biophore activities. A subunit is termed synergistic or biophobic if it could increase or decrease the activity, respectively (Graham et al., 1996; Chakravarti et al., 2012). As such, this package can predict the effective level of skin sensitization for a given compound and has been validated by predicting various adverse effects of drugs, namely carcinogenicity, hepatotoxicity, cardiotoxicity, renal toxicity, and reproductive toxicity. Saiakhov et al. have carried out a pilot study using case Ultra to analyze other adverse effects, including skin sensitization (Saiakhov et al., 2013).

**TABLE 7:** The commercial package and models based on animal, non-animal tests, and mixed test type.

References	Model
Commercial package	
Klopman (1992)	Case
Jaworska et al. (2002)	CATABOL (http://oasis-lmc.org/products/models/environmental-fate-and-ecotoxicity/catabol-301c.aspx)
Dimitrov et al. (2005)	TIMES-SS (http://oasis-lmc.org/products/models/metabolism-simulators/skin-metabolism.aspx)
Kostal and Voutchkova-Kostal (2016)	CADRE-SS
Models based on animal tests	
Enoch et al. (2008)	ToxTree
Barratt et al. (1994), Langton et al. (2006)	Derek Nexus
Wilm et al. (2018)	CAESAR (http://www.caesar-project.eu/index.php?page=results&section=endpoint&ne=2)
Benfenati et al. (2013)	VEGA (https://www.vegahub.eu/)
Toropova and Toropov (2017)	CORAL (http://www.insilico.eu/coral)
Models based on non-animal tests	
Otsubo et al. (2017)	binary classifier based on KeratinoSens™ and h-CLAT
Asturiol et al. (2016)	qualitative skin sensitization predictive model using DT by combining DPRA, KeratinoSens™, and h-CLAT
Roberts and Patlewicz (2018)	Build the model based on DT by combining DPRA, h-CLAT
Models based on mix test type	
Tung et al. (2018); Tung et al. (2019)	*SkinSensPred* (https://cwtung.kmu.edu.tw/skinsensdb/predict)
Borba et al. (2020a), Braga et al. (2017)	*Pred-skin* (http://predskin.labmol.com.br/)
Ohtake et al. (2018)	model based on *in silico* Derek Nexus, *in chemico* DPRA, and *in vitro* h-CLAT
Zang et al. (2017)	DPRA, h-CLAT, KeratinoSens™ results and six physicochemical properties of compounds
Wilm et al. (2020)	Skin Doctor CP (https://nerdd.zbh.uni-hamburg.de/skinDoctorII/)
Borba et al. (2020a)	https://stoptox.mml.unc.edu/

A non-sensitizer might be converted into a sensitizer through a biodegradation metabolism pathway (Jaworska et al., 2002). CATABOL (http://oasis-lmc.org/products/models/environmental-fate-and-ecotoxicity/catabol-301c.aspx) is an online package that can simulate the metabolic pathways of chemicals by predicting the abiotic molecular transformation and enzyme-mediated reactions such as reductive, hydrolytic, oxidative, redox, conjugative reactions, reactions with skin protein, as well as predicting the chemical transformation through spontaneous reactions, enzyme-catalyzed metabolism reactions, and reactions with protein nucleophiles (Jaworska et al., 2002). The tissue metabolism simulator (TIMES) model based on the prediction from CATABOL consists of simulators: 1) using the microbial metabolism simulator to generate the metabolic maps from the training samples; 2) evaluation of skin sensitization potential in light of the metabolic maps and structural alerts (Dimitrov et al., 2005). The TIMES model for skin sensitization (TIMES-SS) package is commercially available and the information about skin metabolism associated with skin sensitization is available online (http://oasis-lmc.org/products/models/metabolism-simulators/skin-metabolism.aspx). The training samples were excerpted from LLNA, GMPT, and human datasets (Dimitrov et al., 2005; Mekenyan et al., 2012). Ivanova et al. expanded the development of the kinetic component into the TIMES-SS model in 2020. In this model, they tried to implement the kinetic of biotic transformations to predict the skin sensitization potential. The initial predictions were consistent with the experimental data for those tested compounds (Ivanova et al., 2020).

The skin sensitization activity of a chemical will also depend on the transformation ability from prohapten into hapten in that the sensitizers themselves are not electrophilic *per se*. Nevertheless, they can undergo enzymatic or oxidative processes to become electrophilic that, in turn, can facilitate the interaction with skin protein, producing antigens consequently (Aptula et al., 2005). Unlike the other packages, Computer Aided Discovery and Redesign-Skin Sensitization (CADRE-SS) is focused on such biological transformation and is comprised of three modules to analyze the reaction in each step: I) skin permeability; II) haptenation and hapten-activation mechanisms, and III) conjugation with protein. The interaction potential between chemicals and skin protein is analyzed by module II using the Smiles ARbitary Target Specification (SMARTS) pattern structure, and compounds are subjected to further analysis by module III once the chemicals are identified as potential haptens. The key event in this process is the adduct formation between the chemical and the Keap1 protein, which contains highly reactive cysteine and lysine amino acids (Kostal and Voutchkova-Kostal, 2016).

SMARTS patterns have been mined by *ToxTree* (Enoch et al., 2008) to identify the potential of skin sensitization. A series of SMARTS patterns based on the previously identified mechanisms of action have been identified, namely aromatic nucleophilic substitution (SNAr), Schiff base formation (SB), Michael-type addition (MA), aliphatic nucleophilic substitution (SN2), and acylation (Ac) (Aptula et al., 2005), in which the covalent bond can be formed between skin protein and sensitizer (Enoch et al., 2008; Enoch et al., 2011). Totally, 104 structural alerts were issued in 2011 (Enoch et al., 2011) and the most updated version is commercially available at https://www.daylight.com/products/toolkit.html through Daylight Toolkit.

Deductive estimation of risk from existing knowledge (DEREK) is an expert knowledge system-based commercial predictive package, in which the structure alerts are proposed to predict the binding potential between electrophilic chemicals and skin protein. Initially, only 40 structure-activity rules for skin sensitization were issued in 1994 (Barratt et al., 1994), and that number increased up to 70 in 2006 (Langton et al., 2006) from the GPMT input data. The modified version of Derek Nexus was released in 2017 using the LLNA EC3 value from over 650 compounds in the Lhasa EC3 dataset (https://www.lhasalimited.org/products/skin-sensitization-assessment-using-derek-nexus.htm) instead of GPMT, which was used in the previous versions. This version features the qualitative prediction for mammalian skin sensitization and the quantitative EC3 prediction for skin sensitizers (Canipa et al., 2017). The skin sensitization structure alerts in Derek Nexus increased from 73 to 90 between 2014 and 2018 and the performance was validated against a dataset over 2,500 chemicals with LLNA and/or GPMT data (Chilton et al., 2018).

### Models Based on Animal Tests

Computer Assisted Evaluation of Industrial Chemical Substances According to Regulations (CAESAR) was developed according to the QSAR validation principles issued by OECD. This model was built by the EU (Cassano et al., 2010; Wilm et al., 2018) and is freely available (http://www.caesar-project.eu). CAESAR can be used to develop QSAR models for five endpoints, namely skin sensitization, carcinogenicity, mutagenicity, bioconcentration factor, and developmental toxicity. The CAESAR model for skin sensitization was derived based on 209 compounds excerpted from a previous study (Gerberick et al., 2005) to classify compounds into sensitizer or non-sensitizer (http://www.caesar-project.eu/index.php?page=results&section=endpoint&ne=2). Afterward, Virtual models for property Evaluation of chemicals within a Global Architecture (VEGA) derived from CAESAR model can predict skin sensitization based on the LLNA data. This binary classifier, which is freely accessible, can be downloaded at http://www.vega-qsar.eu (Benfenati et al., 2013) and the latest version can be found at https://www.vegahub.eu/. Fitzpatrick *et al.* compared the performance of VEGA, TIME-SS, and Derek Nexus in skin sensitization by applying 1,249 substances from the eChemportal skin sensitization dataset (http://www.echemportal.org/echemportal/index.action) and 515 substances from the Interagency Center for the Evaluation of Alternative Toxicological Methods (NICEA™) LLNA database (https://ntp.niehs.nih.gov/pubhealth/evalatm/test-method-evaluations/immunotoxicity/index.html). The results showed that the accuracy of any expert models was about 65%, especially with the substances that were within the application domain of TIME-SS, the accuracy could be reached to 79 and 82% for both datasets (Fitzpatrick et al., 2018). This comparison, in fact, is consistent with the observation made by Teubner et al., in which it has been demonstrated that TIME-SS executed better than the others such as VEGA and DEREK (Teubner et al., 2013). Another model developed by Istituto di Ricerche Farmacologiche Mario Negri (IRCCS) and the Joint Research Center (JRC) is available at https://www.vegahub.eu/. The model is built based on decision trees using 8 descriptors, which are listed at https://www.vegahub.eu/vegahub-dwn/qmrf/QMRF_SKIN_JRC.pdf. The endpoint of this model is skin sensitization on mice (LLNA). When applied to external validation, this model can obtain the accuracy, specificity and sensitivity of 71, 82 and 65%, respectively. All 75 *in silico* models imbedded in VEGA have been implemented into the OECD QSAR Toolbox.

The Correlation and Logic (CORAL) package has been used as a tool for QSAR analyses (Toropov et al., 2013). Afterward, this software was used to develop a tool to predict the skin sensitization (Toropova and Toropov, 2017). Various QSAR models were built based on 204 compounds with the local lymph node assay results using the Monte Carlo technique. The hybrid descriptors calculated via the representation of the molecular structure by SMILES with molecular graph were used to generate the models. The model is available at http://www.insilico.eu/coral.

Very recently, the first ternary predictive model has been developed by Wilm et al. termed Skin Doctor CP (available at: https://nerdd.zbh.uni-hamburg.de/skinDoctorII/) based on the LLNA database. The most distinguishing characteristic of this model is that compounds are initially categorized into sensitizers or non-sensitizers by the first classifier and the predicted sensitizers are further grouped into weak to moderate sensitizers and strong to extreme sensitizers by a second classifier. The model showed the accuracies of 0.90 and 0.73 as well as the efficiencies of 0.42 and 0.90 at the significance levels of 0.10 and 0.30, respectively. However, this ternary classifier did not achieve good performance since the validity values were 0.70, 0.58, and 0.63 for non-sensitizers, weak to moderate sensitizers, and strong to extreme sensitizers, respectively, at the significance level of 0.30 (Wilm et al., 2020).

### Models Based on Non-animal Tests

Unlike the other predictive models, which rely on a single data type, some predictors make decisions based on multiple data types. For instance, Otsubo et al. have built a binary classifier based on KeratinoSens™ and h-CLAT, and chemicals are designated as skin sensitizers if they have positive results by either one of the assays and non-sensitizers otherwise. The predictions produced the sensitivity values of 93.4 and 94.4% as compared with the LLNA and human data, respectively (Otsubo et al., 2017). After collecting data from three non-animal assays, namely DPRA, KeratinoSens™, and h-CLAT, this study was further extended to build a majority voting system. Compounds were defined as sensitizers when at least two positive responses were obtained from those three assays. The accuracy obtained from this model was 90% when compared with human data, whereas that resulted from LLNA alone was merely about 80% as compared with the human data (Urbisch et al., 2015). According to this, multiple data type models can execute better than their single-data-type counterparts.

Asturiol *et al.* took a different approach to develop a qualitative skin sensitization predictive model using decision tree (DT) (Asturiol et al., 2016). The model was derived by combining 3 non-animal test data types, namely DPRA, KeratinoSens™, and h-CLAT. The accuracy of the model was defined by comparing with the LLNA classification (sensitizer/non-sensitizer). The model showed 93% accuracy, 98% sensitivity, and 85% specificity for 269 chemicals (Asturiol et al., 2016). A different approach was taken to build various DTs based on non-animal test results, in which compounds were classified as sensitizers when DPRA gave rise to positive results. Further evaluation by h-CLAT was carried out once compounds were considered as negative by DPRA. Compounds were classified as skin sensitizers if h-CLAT showed positive results, whereas compounds were labeled as non-sensitizers otherwise (Roberts and Patlewicz, 2018). Additionally, various models were developed according to binary combinations of those three non-animal tests, namely DPRA, h-CLAT, and KeratinoSens™. It was found that the combination of DPRA and h-CLAT performed best in distinguishing sensitizers from non-sensitizers. More importantly, it was observed that all of the models based on combinations of non-animal tests usually performed better than their counterparts based on a single test that, actually, is consistent with the previous observation (vide supra) (Urbisch et al., 2016; Otsubo et al., 2017), suggesting that predictive models based on the single non-animal test are not sufficient to comprehensively render the skin sensitization complicated process (Adler et al., 2011; Hartung et al., 2011; Wilm et al., 2018; Madden et al., 2020).

### Models Based on Mixed Test Types

Most of the published packages or models are binary classification systems, *viz*. sensitizer *vs*. non-sensitizer, based on one or more than one non-animal tests. Integrated approaches to testing and assessment (IATA) has taken a different approach by combining various animal tests, non-animal tests, and *in silico* models to predict the latency of skin sensitization (OECD, 2016a). IATA includes the models, which are flexible and non-formalized judgment based, e.g. grouping and read-across or more structured, rule based approaches such as Integrated Testing Strategy (ITS) (OECD, 2016a). ITS can combine DPRA, KeratinoSens™, and h-CLAT (Jaworska et al., 2015; Urbisch et al., 2015), DPRA, SENS-IS and/or h-CLAT (Clouet et al., 2017), two of 3 non-animal tests, namely DPRA, KeratinoSens™, and h-CLAT, to generate the predictive model (Otsubo et al., 2017) based on *in chemico*, *in vitro*, and *in silico* data (Jaworska et al., 2011). It has been found that the models based on this strategy showed better performance. For instance, this approach was implemented to develop various models based on the combinations of 2 or 3 non-animal datasets, namely DPRA, KeratinoSens™, and h-CLAT and the built model with the selection of 3 non-animal datasets displayed the highest sensitivity and yet the lowest specificity as compared with its counterparts with the combination of only 2 of 3 non-animal datasets (Otsubo et al., 2017). In 2017, Douglas Connect Integrated Testing Strategy (DC ITS) SkinSens was launched to access the integrated testing strategy developed by Jaworska (Jaworska et al., 2013). The latest updated version for DC ITS SkinSens is SaferSkin™ (https://saferworldbydesign.com/saferskin/).

*SkinSensPred*, which is a skin sensitization predictive function, was developed in 2019 based on *SkinSensDB*, is freely accessed at https://cwtung.kmu.edu.tw/skinsensdb/predict (Tung et al., 2018; Tung et al., 2019). This multitask learning model is based on three AOP key events and human skin sensitization test using protein binding (DPRA), keratinocyte activation, dendritic cell activation to binarily classify results in the human test. This model can analyze the application domain (AD) and structure alerts (SA) to predict the human sensitization potential of a chemical. When applied to novel chemicals within the defined AD, this model could reach an accuracy of 84.3% (Tung et al., 2019). In addition, a majority voting model (2 out of 3) (Urbisch et al., 2015) and a DT model (Roberts and Patlewicz, 2018) can be implemented as the read-across predictive methods.

In 2018, Del Bufalo *et al.* developed an alternative integrated testing for skin sensitization using the combination of 3 *in vitro* methods (DPRA, Keratinosens™, U-SENS™), two *in silico* tools (TIMES-SS, TOXTREE) and physicochemical parameters (volatility, pH). These data were run in 5 different classification models (Boosting, Naive Bayes, support vector machine (SVM), Sparse Partial Least Squares Discriminant Analysis, and Expert Scoring). The validation results were used in the stacking meta-model to evaluate the skin sensitization potential (Del Bufalo et al., 2018). The predictions achieved the accuracies of 93 and 91% for the training set and test set, respectively when compared with LLNA hazard data. A larger data set was used to validate this model in 2020 (Tourneix et al., 2020). The Defined Approach (version 5) was used (Tourneix et al., 2020) to evaluate the skin sensitization potential of 219 compounds.

*Pred-skin*, which is accessible at http://predskin.labmol.com.br/, is a consensus Naïve Bayes model that employs multiple QSAR models based on various human, LLNA, and non-animal data to predict skin sensitization. This model exhibited good performance in predicting human skin sensitization with sensitivity (94%) and specificity (84%). When applied to 11 new potential sensitizers, which were not included in the dataset, Pred-skin exerts an efficient approach to identify nine sensitizers (Borba et al., 2020a; Braga et al., 2017).

Zang *et al.* have published an *in silico* model, which was derived by combining the non-animal data, namely DPRA, h-CLAT, and KeratinoSens™, and six physicochemical properties, namely octanol/water partition coefficient, water solubility, vapor pressure, melting point, boiling point, and molecular weight, as the descriptors to predict LLNA and human outcomes (Zang et al., 2017). Compounds were classified into sensitizers or nonsensitizers in this model, and the sensitizers were further divided into 1A (strong) or 1B (weak) sensitizer subcategories based on the GHS. The model achieved the accuracy of 88% for the prediction of LLNA outcomes and 81% prediction for human test outcomes (Zang et al., 2017).

Ohtake et al. have published a predictive model based on highly heterogeneous data, namely *in silico* Derek Nexus, *in chemico* DPRA, and *in vitro* h-CLAT, in which the results of DPRA and h-CLAT were scaled between 0 and 3, and the outcomes from Derek Nexus were reduced between 0 and 1, and the final total score was generated by summing those scores. A compound is defined as a strong sensitizer when its total score is larger than 7, and a weak sensitizer when its total score is between 2 and 6 (Ohtake et al., 2018). The unique characteristic of this model is the fact that the skin sensitizers are further divided into the strong and weak ones in this model despite the fact that this multiple classification system is not the same as the animal or human test classification. However, only nine isocyanates were included and the prediction results indicated that this model underestimated the skin sensitization potential when compared with LLNA data.

In 2020, Silva et al. used the different combinations of *in vitro* (human information)*, in chemico* (DPRA), and/or *in silico* (the formation descriptor calculated by the TIMES-SS) data to build the models, which can predict the skin sensitization potential. The results showed that the combination of *in vitro, in chemico*, and *in silico* achieved the best prediction results. Moreover, the models reached an accuracy of 100% in differentiating sensitizers from non-sensitizers. When the same model was, it exhibited the accuracies of 98.8 and 97.5% accuracy when applied to the compounds based on GHS classification (3-level scales) and human data (6-level scales), respectively (Silva et al., 2020).

### Machine Learning-Based Models

Currently, a variety of simulation approach DT, artificial neural network (ANN), support vector machine (SVM), AdaBoost, the iterative least squares linear discriminant (TILSQ), logistic regression (LR), and K-step yard sampling (KY) method (U.S. Patent No. 7725413) (Kohtarou, 2010), consensus methods, Bayesian networks have been adopted to build various skin sensitization predictive models. The detail of some machine learning schemes has been described and illustrated by a review paper of Tarca et al. and the assessment of some defined approaches in skin sensitization prediction was evaluated by Kleinstreuer et al. (Tarca et al., 2007; Kleinstreuer et al., 2018).

The SH test is designed to measure changes in cell surface thiols on hapten-treated cells and used to develop the first version of ANN-based iSENS to predict skin sensitization with the combination of h-CLAT data (Suzuki et al., 2009; Hirota et al., 2013). The second version was released afterward using the combination of the ARE assay data and *n*-octanol‒water partition coefficient (log *p*) value (Natsch and Emter, 2008; Tsujita-Inoue et al., 2014). Further extended versions of the ANN model were based on various combinations of h-CLAT, DPRA, KeratinoSens™, and SH test (Hirota et al., 2015). It has been observed that the performance of an ANN model actually depended on the combination of data types. For instance, the ANN model based on the combination of h-CLAT and DPRA showed a better correlation with LLNA than other combinations such as DPRA and ARE assay or the SH test and ARE assay. The predictive models based on three descriptors such as the selection of h-CLAT, DPRA, and ARE assay or h-CLAT, SH test, and ARE assay produced higher correlation coefficients, *viz*. *r* values, and smaller prediction errors than their two-data-type counterparts (Hirota et al., 2015).

More recently, Macmillan and Chilton have combined Derek Nexus and non-animal KeratinoSens™, h-CLAT, DPRA, and U-SENS™ tests to develop a DT model. The derived DT model showed great performance with 73 and 76% accuracy of LLNA and human data, respectively, depending on the GHS classification (Macmillan and Chilton, 2019). A variety of machine learning-based schemes, namely ANN, SVM, AdaBoost, and TILSQ, were employed to build skin sensitization predictive models based on linear and non-linear discriminant analyses of 291 samples. It was found that SVM and AdaBoost models based on 32 descriptors to encode the 2-D and 3-D structural characteristics showed the highest performance with 100% accuracy of negative and positive (Sato et al., 2009). This investigation was further extended by including more samples (593 compounds) and adopting a novel KY scheme (Sato et al., 2012). Unlike any binary classification models, compounds were allotted to negative, positive, and gray zones through multiple steps in this study. Compounds in the gray zone, which was a confusing area, were repeatedly deposited into the positive and negative zones until no compound was left, the strategy was illustrated by Sato et al. in 2012. All 593 compounds were classified impeccably in 3 steps (Sato et al., 2012).

Strickland et al. have adopted the LR and SVM schemes to develop predictive models based on non-animal tests, namely DPRA, h-CLAT, and KeratinoSens™ using 6 physicochemical properties, namely log *p*, water solubility, vapor pressure, melting point, boiling point, and molecular weight. It was found that log *p* was the most pivotal factor in determining skin sensitization among various physicochemical properties that was further assured by Gleeson et al. (Gleeson and Gleeson, 2020). Of various combinations of non-animal test data, models that included the combination of DPRA and h-CLAT produced the highest accuracy (Strickland et al., 2017). Pre- and pro-hapten sensitizers, which need to go through chemical transformation through air exposure (Karlberg et al., 1992; Sköld et al., 2002) or metabolism pathway (Nilsson et al., 2005; van Eijl et al., 2012) prior to the sensitization process, hinder the accuracy of current *in vitro* assays. Accordingly, a novel tri-culture assay system, which includes MUTZ-3-derived Langerhans cells, HaCaT keratinocytes, and primary dermal fibroblasts, and then measures the secretion levels of cytokines after these cells are exposed to test compounds, viz. sensitizer or non-sensitizer, has been proposed. Numerous SVM models were developed based on the stimulation indices (SI) of 27 human cytokines, namely IL-1β, IL-1ra, IL-2, IL-4, IL-5, IL-6, IL-7, IL-8, IL-9, IL-10, IL-12, IL-13, IL-15, IL-17, eotaxin, basic FGF, G-CSF, GM-CSF, IFN-γ, IP-10, MCAF, MIP-1α, MIP-β, PDGF-BB, RANTES, TNF-α, and VEGF to identify the most significant cytokines associated with skin sensitization. It was observed that the SVM model based on the top three ranking biomarkers, namely IL-8, MIP-1β, and GM-CSF, in tri-culture assay showed the highest performance with the prediction accuracy of 91%, and the detection of pre- and pro-hapten was improved accordingly (Lee et al., 2018).

Matsumura et al. perfomed a study using QSAR-like deep neural network (DNN) and light gradient boosting machine (LightGBM) to evaluate the potential of skin sensitization. Physical and structural properties of chemicals and the skin sensitizer/non-sensitizers based on the classification of GHS were used as input variables. The results showed that the dual-input LightGBM model (74%) and dual-DNN model (72%) were moderately accurate when compared with the traditional approaches (Matsumura, 2020).

In addition, other algorithms and methods have been adopted to improve the classification performance. For instance, Abdallh *et al* used binary crow search algorithm (BCSA) that was initially proposed by Askarzadeh in 2016 (Askarzadeh, 2016) to select the most relevant descriptors in the model development. The results gained the classification accuracy for the compound into sensitizer/non-sensitizer.

## Future Perspectives

The dermatology researched has shifted into a new paradigm after the introduction of artificial intelligence. Most of the applications involve in image analysis, but also include the analyses of the physiochemical properties of substances (Gomolin et al., 2020). Various applications in toxicity and environmental hazard endpoints, for instance, indicate that the great diversity of QSAR models (Chinen and Malloy, 2020). Data quality plays a critical role in model development and it is almost impossible to build a sound *in silico* model based on contaminated or impure data, especially for the quantitative predictive models (Cherkasov et al., 2014). The accuracy of a virtual model depends on the data quality, the lack of instruction of how to report and publish the toxicogenomic studies can hinder the usage of data in building the computational models (FitzGerald, 2020). There are some strategies to build an *in silico* model. The first strategy is to collect a large number of experimental data, extended the data coverage, and the use of big data approach. Another more effective way to build a model relied on deeply understanding the biological mechanism to predict the biochemical processes and the bioactivity of the novel compounds. The model based on mechanism does not need a large amount of training set, but it needs highly accurate experimental data (Kostal and Voutchkova-Kostal, 2020). Accordingly, it is of necessity to implement data curation prior to model development by removing those assay data obtained from impurity or mixture to maintain data integrity.

In 2020, Golden et al. carried out an investigation, which compared the accuracy of eight *in silico* models (PredSkin, Toxtree, QSAR Toolbox, Danish QSAR database, CAESAR, REACHAcross™, TIMES-S and Derek Nexus) against human data sets. Most of the models showed the accuracies of 70–80% on human data sets, suggesting that *in silico* models can be a convenient and inexpensive tool to define the skin sensitization in human (Golden et al., 2021). There is no doubt that an *in silico* model to predict skin sensitization based on human data will be more realistic and much needed. However, the scarcity in consistent human data in the public domain has created an unsurmountable hurdle for creating a sound predictive model due to their small amount of available data and limited structural diversity (Cherkasov et al., 2014). The relatively ample amount of LLNA data makes it a better alternative since the LLNA predictions can well correlate with the human tests in most of the cases and it has been commonly recognized as the gold standard for the human skin sensitization. However, the LLNA model is inevitably susceptible to some chemotypes, suggesting that it is of necessity to develop different predictive models for different chemotypes to accommodate the variations in skin sensitization mechanism. Some problems remain unresolved when the test compounds have consisted of more than one chemotype or when the test compounds lie outside of the applicability domain of the derived model. With the effort to improve the accuracy of skin sensitization tests, Leontaridou et al. identified the borderline range (BR) around the classification threshold of DPRA, LuSens, h-CLAT and LLNA. The substances with the test results fell into the BR and another available test method was required to depict the positive/negative outcome (Leontaridou et al., 2017).

The applications of animal tests on cosmetics products have been prohibited in Europe since 2013 (European Commission, 2013a) and lately, some countries also have accepted the OECD non-animal method to test the skin sensitization (Strickland et al., 2019). Nevertheless, animal tests, especially GMPT and LLNA, are still available and required by numerous countries such as Canada, China, Brazil, Japan, and the United States (Daniel et al., 2018). Additionally, the applications of animal tests for pesticides, plant protection products, pharmaceuticals, household products, art materials, industrial chemicals, medical devices, and workplace chemicals are needed and still acceptable in many industries, even in Europe (Daniel et al., 2018). These data provide valuable resources for building some *in silico* models to assess the covert of skin sensitization. In addition, the skin sensitization QSAR models can be applied to not only cosmetic ingredients but also the compound, which can impact the ecosystems such as dye pollution, the effect of personal care products on aquatic species, or plasticizers (Arulanandam et al., 2021; Funar-Timofei and Ilia, 2020; Khan et al., 2020) as well as the pharmacokinetics profiles of low molecular weight oligo-hydroxyalkanoates (Roman et al., 2020), suggesting that the skin sensitization models has a wide range of applications. To date, most of the published skin sensitization models are qualitative predictions, *viz*. binary classification of sensitizers or non-sensitizers. Nevertheless, it has been observed that a quaternary predictive model would execute better than its ternary counterpart, which, in turn, performed better than a binary one in the case of drug-induced liver injury (DILI) prediction (Weng and Leong, 2020). Accordingly, it is plausible to expect a multiple-class qualitative model to predict skin sensitization can function better than a two-class one. Predictive models based on a single type of assay data can only take into account one single pathway, suggesting that no single non-animal test can comprehensively render the whole complex skin sensitization process (Adler et al., 2011; Hartung et al., 2011). Additionally, some cosmetic or commercial products have currently contained sensitizers (Robinson et al., 2000) despite the fact that they do not trigger adverse reactions in the induction and elicitation phases when the applied doses are low. In addition, the application volume of those cosmetic products that are in direct and persistent contact with skin, e.g. cream or foundation, are different from those that can be washed or rinsed off, e.g. shampoo or body lotion. The differences in exposure dose between these groups can be up to 30 folds (Fewings and Menné, 1999; Frosch et al., 1995; Robinson et al., 2000). Therefore, qualitative *in silico* or non-animal models have hindered applications for those weak or moderate sensitizers in pharmaceuticals or cosmeceuticals markets, more importantly, a quantitative prediction model can be truly useful. To solve this problem, the package *SpheraCosmolife*, which is implemented in VEGAHUB, can process various ingredients in a product, has been derived recently. It can predict the mutagenicity, genotoxicity, and skin sensitization based on the concentration and the product type, namely lotion, shampoo, shower gel, *etc*, recorded in the internal database (Regulation (EC) No. 1223/2009 of the European Parliament and of the Council of 30 November 2009 on cosmetic products). This software is available at https://www.vegahub.eu/download/sphera-cosmolife-download/and is implemented in VEGA. However, some challenges still remain since they cannot predict the skin sensitization caused by metals (Biswas et al., 2020).

Based on the principles published by the International Cooperation on Cosmetic Regulation (ICCR), Gilmour et al. displayed next generation risk assessment (NGRA) framework for skin sensitizers in 2020, which can be illustrated in Figure 1 of their publication (Gilmour et al., 2020). According to four elements of risk assessment, which included consumer exposure, hazard identification, hazard characterization and establishment of a dose response, that they presented a workflow assembled three tires and integrated all relevant information using a weight of evidence approach to predict a chemical to be a skin sensitizer or non-sensitizer (Gilmour et al., 2020).

The number of *in silico* models to predict skin sensitization has increased for the past few years. Those models have been built based on *in vivo, in vitro, in chemico*, and/or *in silico* data. Johnson *et al.* defined the rules and principles to develop the effective *in silico* skin sensitization models to facilitate the implementation and acceptance of *in silico* approaches based on the skin sensitization mechanism and the strengths/limitations of each experimental methods. The standardization of this hazard assessment framework has further strengthened the use and application of *in silico* tools in agencies and industries (Johnson et al., 2020).

With the effort to collect the available web portal to predict 6 of acute toxicity tests, namely acute oral toxicity, acute dermal toxicity, acute inhalation toxicity, skin irritation and corrosion, eye irritation and corrosion, and skin sensitization), Borba et al. developed a package called Systemic and Topical chemical Toxicity (STopTox), which is available at https://stoptox.mml.unc.edu/ (Borba et al., 2020b).

## Conclusion

*In vitro* skin sensitization tests alone cannot replace human and animal tests because they only focus on one single pathway in AOP. *In silico* approach, conversely, has more advantages than *in vitro* tests since it can take into account more than one AOP key event by combining various *in chemico*, *in vitro*, and *in vivo* data simultaneously. To date, most of the published *in silico* models only classify chemicals into sensitizers or non-sensitizer. This binary classification system has severely limited the applications of weak or moderate sensitizers in commercial products. Multiple-class *in silico* models can be greatly useful in practical applications as exemplified by the DILI study (*vide supra*) and quantitative ones will be even better. With the effort to develop the quantitative models, some *in silico* models have been generated recently, and yet they inevitability suffer from major limitations, suggesting that these quantitative models still need to be improved to be more accurate and have a wider range of applications in evaluating the skin sensitization potential. The development of robust and accurate *in silico* models for skin sensitization prediction is still a long and winding path ahead of the molecular modeling community.

## References

[B1] Adler S. Basketter D. Creton S. Pelkonen O. van Benthem J. Zuang V. (2011). Alternative (Non-animal) Methods for Cosmetics Testing: Current Status and Future Prospects-2010. Arch. Toxicol. 85 (5), 367–485. 10.1007/s00204-011-0693-2 21533817

[B2] Ahlfors S. R. Sterner O. Hansson C. (2003). Reactivity of Contact Allergenic Haptens to Amino Acid Residues in a Model Carrier Peptide, and Characterization of Formed Peptide-Hapten Adducts1. Skin Pharmacol. Physiol. 16 (1), 59–68. 10.1159/000068288 12566830

[B3] Alkilani A. McCrudden M. T. Donnelly R. (2015). Transdermal Drug Delivery: Innovative Pharmaceutical Developments Based on Disruption of the Barrier Properties of the Stratum Corneum. Pharmaceutics 7 (4), 438–470. 10.3390/pharmaceutics7040438 26506371 PMC4695828

[B4] Api A. M. Basketter D. Lalko J. (2015). Correlation between Experimental Human and Murine Skin Sensitization Induction Thresholds. Cutan. Ocul. Toxicol. 34 (4), 298–302. 10.3109/15569527.2014.979425 25430073

[B5] Aptula A. O. Patlewicz G. Roberts D. W. (2005). Skin Sensitization: Reaction Mechanistic Applicability Domains for Structure−Activity Relationships. Chem. Res. Toxicol. 18 (9), 1420–1426. 10.1021/tx050075m 16167834

[B6] Arulanandam C. D. Prathiviraj R. Kaveriyappan G. R. (2021). Raspberry Pi: Assessments of Emerging Organic Chemicals by the Predictive In Silico Methods. bioRxiv 2001 (2015), 426465. 10.1101/2021.01.15.426465

[B7] Ashikaga T. Sakaguchi H. Sono S. Kosaka N. Ishikawa M. Nukada Y. (2010). A Comparative Evaluation of *In Vitro* Skin Sensitisation Tests: the Human Cell-Line Activation Test (H-CLAT) *versus* the Local Lymph Node Assay (LLNA). Alternatives Lab. Anim. 38 (4), 275–284. 10.1177/026119291003800403 20822320

[B8] Askarzadeh A. (2016). A Novel Metaheuristic Method for Solving Constrained Engineering Optimization Problems: Crow Search Algorithm. Comput. Structures 169, 1–12. 10.1016/j.compstruc.2016.03.001

[B9] Asturiol D. Casati S. Worth A. (2016). Consensus of Classification Trees for Skin Sensitisation Hazard Prediction. Toxicol. Vitro 36, 197–209. 10.1016/j.tiv.2016.07.014 27458072

[B10] Ball N. Cagen S. Carrillo J.-C. Certa H. Eigler D. Emter R. (2011). Evaluating the Sensitization Potential of Surfactants: Integrating Data from the Local Lymph Node Assay, guinea Pig Maximization Test, and *In Vitro* Methods in a Weight-Of-Evidence Approach. Regul. Toxicol. Pharmacol. 60 (3), 389–400. 10.1016/j.yrtph.2011.05.007 21645576

[B11] Barratt M. D. Basketter D. A. Chamberlain M. Payne M. P. Admans G. D. Langowski J. J. (1994). Development of an Expert System Rulebase for Identifying Contact Allergens. Toxicol. Vitro 8 (4), 837–839. 10.1016/0887-2333(94)90081-7 20693025

[B12] Basketter D. A. Alépée N. Ashikaga T. Barroso J. Gilmour N. Goebel C. (2014). Categorization of Chemicals According to Their Relative Human Skin Sensitizing Potency. Dermatitis : Contact atopic, Occup. Drug 25 (1), 11–21. 10.1097/der.0000000000000003 24407057

[B13] Bauch C. Kolle S. N. Fabian E. Pachel C. Ramirez T. Wiench B. (2011). Intralaboratory Validation of Four *In Vitro* Assays for the Prediction of the Skin Sensitizing Potential of Chemicals. Toxicol. Vitro 25 (6), 1162–1168. 10.1016/j.tiv.2011.05.030 21669280

[B14] Bauch C. Kolle S. N. Ramirez T. Eltze T. Fabian E. Mehling A. (2012). Putting the Parts Together: Combining *In Vitro* Methods to Test for Skin Sensitizing Potentials. Regul. Toxicol. Pharmacol. 63 (3), 489–504. 10.1016/j.yrtph.2012.05.013 22659254

[B15] Benfenati E. Manganaro A. Gini G. (2013). “VEGA-QSAR: AI Inside a Platform for Predictive Toxicology,” in Proceedings of the workshop “Popularize Artificial Intelligence 2013”. Turin, 1107.

[B16] Bezerra S. F. Santos Rodrigues B. Silva A. C. G. Ávila R. I. Brito H. R. G. Cintra E. R. (2021). Application of the Adverse Outcome Pathway Framework for Investigating Skin Sensitization Potential of Nanomaterials Using New Approach Methods. Contact Dermatitis 84 (2), 67–74. 10.1111/cod.13669 32683706

[B17] Biswas R. Akermi S. Jayant S. (2020). Prediction Of Skin Sensitization By *In-Silico* Tools: Today and Future. J. Sci. 3 (3), 13–19.

[B18] Borba J. V. B. Braga R. C. Alves V. M. Muratov E. N. Kleinstreuer N. Tropsha A. (2020a). Pred-Skin: A Web Portal for Accurate Prediction of Human Skin Sensitizers. Chem. Res. Toxicol. 34, 258–267. 10.1021/acs.chemrestox.0c00186 32673477

[B19] Borba J. V. V. B. Alves V. Braga R. Korn D. Overdahl K. Silva A. C. (2020b). STopTox: An In-Silico Alternative to Animal Testing for Acute Systemic and TOPical TOXicity. ChemRxiv. 10.26434/chemrxiv.13283930.v1 PMC8863177 35192406

[B20] Braga R. C. Alves V. M. Muratov E. N. Strickland J. Kleinstreuer N. Trospsha A. (2017). Pred-Skin: A Fast and Reliable Web Application to Assess Skin Sensitization Effect of Chemicals. J. Chem. Inf. Model. 57 (5), 1013–1017. 10.1021/acs.jcim.7b00194 28459556

[B21] Canipa S. J. Chilton M. L. Hemingway R. Macmillan D. S. Myden A. Plante J. P. (2017). A Quantitative *In Silico* Model for Predicting Skin Sensitization Using a Nearest Neighbours Approach within Expert-Derived Structure-Activity Alert Spaces. J. Appl. Toxicol. 37 (8), 985–995. 10.1002/jat.3448 28244128

[B22] Cassano A. Manganaro A. Martin T. Young D. Piclin N. Pintore M. (2010). CAESAR Models for Developmental Toxicity. Chem. Cent. J. 4 (Suppl. 1). 10.1186/1752-153x-4-s1-s4 PMC2913331 20678183

[B23] Chakravarti S. K. Saiakhov R. D. Klopman G. (2012). Optimizing Predictive Performance of CASE Ultra Expert System Models Using the Applicability Domains of Individual Toxicity Alerts. J. Chem. Inf. Model. 52 (10), 2609–2618. 10.1021/ci300111r 22947043

[B24] Cherkasov A. Muratov E. N. Fourches D. Varnek A. Baskin I. I. Cronin M. (2014). QSAR Modeling: Where Have You Been? Where Are You Going to?. J. Med. Chem. 57 (12), 4977–5010. 10.1021/jm4004285 24351051 PMC4074254

[B25] Chilton M. L. Macmillan D. S. Steger-Hartmann T. Hillegass J. Bellion P. Vuorinen A. (2018). Making Reliable Negative Predictions of Human Skin Sensitisation Using an *In Silico* Fragmentation Approach. Regul. Toxicol. Pharmacol. 95, 227–235. 10.1016/j.yrtph.2018.03.015 29580972

[B26] Chinen K. Malloy T. (2020). QSAR Use in REACH Analyses of Alternatives to Predict Human Health and Environmental Toxicity of Alternative Chemical Substances. Integr. Environ. Assess. Manag. 16 (5), 745–760. 10.1002/ieam.4264 32162772

[B27] Cho S.-A. An S. Park J.-H. (2019). High-throughput Screening (HTS)-based Spectrophotometric Direct Peptide Reactivity Assay (Spectro-DPRA) to Predict Human Skin Sensitization Potential. Toxicol. Lett. 314, 27–36. 10.1016/j.toxlet.2019.07.014 31295538

[B28] Cho S.-A. Jeong Y. H. Kim J. H. Kim S. Cho J.-C. Heo Y. (2014). Method for Detecting the Reactivity of Chemicals towards Peptides as an Alternative Test Method for Assessing Skin Sensitization Potential. Toxicol. Lett. 225 (1), 185–191. 10.1016/j.toxlet.2013.12.007 24362008

[B29] Clausen B. E. Stoitzner P. (2015). Functional Specialization of Skin Dendritic Cell Subsets in Regulating T Cell Responses. Front. Immunol. 6, 534. 10.3389/fimmu.2015.00534 26557117 PMC4617171

[B30] Clouet E. Kerdine-Römer S. Ferret P.-J. (2017). Comparison and Validation of an *In Vitro* Skin Sensitization Strategy Using a Data Set of 33 Chemical References. Toxicol. Vitro 45, 374–385. 10.1016/j.tiv.2017.05.014 28539215

[B31] Cockshott A. Evans P. Ryan C. A. Gerberick G. F. Betts C. J. Dearman R. J. (2006). The Local Lymph Node Assay in Practice: a Current Regulatory Perspective. Hum. Exp. Toxicol. 25 (7), 387–394. 10.1191/0960327106ht640oa 16898167

[B32] Costin G.-E. Hill E. Brown J. Clippinger A. J. (2020). Qualification of a Non-animal Vaginal Irritation Method Admitted as Nonclinical Assessment Model (NAM) in the Incubator Phase of the United States Food and Drug Administration (US FDA) Medical Devices Development Tool (MDDT). Toxicol. Vitro 62, 104680. 10.1016/j.tiv.2019.104680 31626901

[B33] Cottrez F. Boitel E. Auriault C. Aeby P. Groux H. (2015). Genes Specifically Modulated in Sensitized Skins Allow the Detection of Sensitizers in a Reconstructed Human Skin Model. Development of the SENS-IS Assay. Toxicol. Vitro 29 (4), 787–802. 10.1016/j.tiv.2015.02.012 25724174

[B34] Cottrez F. Boitel E. Ourlin J.-C. Peiffer J.-L. Fabre I. Henaoui I.-S. (2016). SENS-IS, a 3D Reconstituted Epidermis Based Model for Quantifying Chemical Sensitization Potency: Reproducibility and Predictivity Results from an Inter-laboratory Study. Toxicol. Vitro 32, 248–260. 10.1016/j.tiv.2016.01.007 26795242

[B35] Daniel A. B. Strickland J. Allen D. Casati S. Zuang V. Barroso J. (2018). International Regulatory Requirements for Skin Sensitization Testing. Regul. Toxicol. Pharmacol. 95, 52–65. 10.1016/j.yrtph.2018.03.003 29518484 PMC5935556

[B36] de Freitas Silva M. Pruccoli L. Morroni F. Sita G. Seghetti F. Viegas C. (2018). The Keap1/Nrf2-ARE Pathway as a Pharmacological Target for Chalcones. Molecules 23, 1803. 10.3390/molecules23071803 PMC6100069 30037040

[B37] Dean J. H. Twerdok L. E. Tice R. R. Sailstad D. M. Hattan D. G. Stokes W. S. (2001). ICCVAM Evaluation of the Murine Local Lymph Node Assay. Regul. Toxicol. Pharmacol. 34 (3), 258–273. 10.1006/rtph.2001.1497 11754530

[B38] Del Bufalo A. Pauloin T. Alepee N. Clouzeau J. Detroyer A. Eilstein J. (2018). Alternative Integrated Testing for Skin Sensitization: Assuring Consumer Safety. Appl. Vitro Toxicol. 4 (1), 30–43. 10.1089/aivt.2017.0023

[B39] Dimitrov S. D. Low L. K. Patlewicz G. Y. Kern P. S. Dimitrova G. D. Comber M. H. I. (2005). Skin Sensitization: Modeling Based on Skin Metabolism Simulation and Formation of Protein Conjugates. Int. J. Toxicol. 24 (4), 189–204. 10.1080/10915810591000631 16126613

[B40] Doke S. K. Dhawale S. C. (2015). Alternatives to Animal Testing: A Review. Saudi Pharm. J. 23 (3), 223–229. 10.1016/j.jsps.2013.11.002 26106269 PMC4475840

[B41] Emter R. Ellis G. Natsch A. (2010). Performance of a Novel Keratinocyte-Based Reporter Cell Line to Screen Skin Sensitizers *In Vitro* . Toxicol. Appl. Pharmacol. 245 (3), 281–290. 10.1016/j.taap.2010.03.009 20307559

[B42] Enoch S. J. Ellison C. M. Schultz T. W. Cronin M. T. D. (2011). A Review of the Electrophilic Reaction Chemistry Involved in Covalent Protein Binding Relevant to Toxicity. Crit. Rev. Toxicol. 41 (9), 783–802. 10.3109/10408444.2011.598141 21809939

[B43] Enoch S. J. Madden J. C. Cronin M. T. D. (2008). Identification of Mechanisms of Toxic Action for Skin Sensitisation Using a SMARTS Pattern Based Approach. SAR QSAR Environ. Res. 19 (5-6), 555–578. 10.1080/10629360802348985 18853302

[B44] European Commission. (2013a). Communication from the Commission to the European Parliament and the Council on the Animal Testing and Marketing Ban and on the State of Play in Relation to Alternative Methods in the Field of Cosmetics.

[B45] European Commission. (2013b). On the Animal Testing and Marketing Ban and on the State of Play in Relation to Alternative Methods in the Field of Cosmetics, Communication from the Commission to the European Parliament and the Council.

[B46] European Union (2003). Directive 2003/15/EC of the European Parliament and of the Council of 27 February 2003 Amending Council Directive 76/768/EEC on the Approximation of the Laws of the Member States Relating to Cosmetic Products. (Brussels: Official Journal of the European Union).

[B47] Fewings J. Menné T. (1999). An Update of the Risk Assessment for Methylchloroisothiazolinone/methylisothiazolinone (MCI/MI) with Focus on Rinse-Off Products. Contact Dermatitis 41 (1), 1–13. 10.1111/j.1600-0536.1999.tb06200.x 10416701

[B48] FitzGerald R. E. (2020). Adverse Outcome Pathway Bridge Building from Research to Regulation. Chem. Res. Toxicol. 33 (4), 849–851. 10.1021/acs.chemrestox.9b00527 32186379

[B49] Fitzpatrick J. M. Roberts D. W. Patlewicz G. (2018). An Evaluation of Selected (Q)SARs/expert Systems for Predicting Skin Sensitisation Potential. SAR QSAR Environ. Res. 29 (6), 439–468. 10.1080/1062936X.2018.1455223 29676182 PMC6077848

[B50] Frosch P. J. Lahti A. Hannuksela M. Andersen K. E. Wilkinson J. D. Shaw S. (1995). Chloromethylisothiazolone/methylisothiazolone (CMI/MI) Use Test with a Shampoo on Patch-Test-Positive Subjects Results of a Multicentre Double-Blind Crossover Trial. Contact Dermatitis 32 (4), 210–217. 10.1111/j.1600-0536.1995.tb00671.x 7600776

[B51] Fujita M. Yamamoto Y. Watanabe S. Sugawara T. Wakabayashi K. Tahara Y. (2019). Cause of and Countermeasures for Oxidation of the Cysteine-Derived Reagent Used in the Amino Acid Derivative Reactivity Assay. J. Appl. Toxicol. 39 (2), 191–208. 10.1002/jat.3707 30221369

[B52] Funar-Timofei S. Ilia G. (2020). “QSAR Modeling of Dye Ecotoxicity,” Ecotoxicological QSARs. Editor Roy K. (New York, NY: Springer US), 405–436. 10.1007/978-1-0716-0150-1_18

[B53] Gäfvert E. Shao L. P. Karlberg A.-T. Nilsson U. Nilsson J. L. G. (1994). Contact Allergy to Resin Acid Hydroperoxides. Hapten Binding via Free Radicals and Epoxides. Chem. Res. Toxicol. 7 (2), 260–266. 10.1021/tx00038a020 8199316

[B54] Gefen T. Vaya J. Khatib S. Rapoport I. Lupo M. Barnea E. (2015). The Effect of Haptens on Protein-Carrier Immunogenicity. Immunol. 144 (1), 116–126. 10.1111/imm.12356 PMC4264915 25041614

[B55] Gerberick F. Aleksic M. Basketter D. Casati S. Karlberg A.-T. Kern P. (2008). Chemical Reactivity Measurement and the Predictive Identification of Skin Sensitisers. Alternatives Lab. Anim. 36 (2), 215–242. 10.1177/026119290803600210 18522487

[B56] Gerberick F. G. Robinson M. K. Ryan C. A. Dearman R. J. Kimber I. Basketter D. A. (2001). Contact Allergenic Potency: Correlation of Human and Local Lymph Node Assay Data. Am. J. Contact Dermatitis 12 (3), 156–161. 10.1053/ajcd.2001.2392610.1097/01634989-200109000-00005 11526521

[B57] Gerberick G. F. Ryan C. A. Dearman R. J. Kimber I. (2007a). Local Lymph Node Assay (LLNA) for Detection of Sensitization Capacity of Chemicals. Methods 41 (1), 54–60. 10.1016/j.ymeth.2006.07.006 16938465

[B58] Gerberick G. F. Ryan C. A. Kern P. S. Schlatter H. Dearman R. J. Kimber I. (2005). Compilation of Historical Local Lymph Node Data for Evaluation of Skin Sensitization Alternative Methods. Dermatitis (Am. J. Contact Dermat.) 16 (4), 157–202. 10.2310/6620.2005.05040 16536334

[B59] Gerberick G. F. (2016). The Use of Peptide Reactivity Assays for Skin Sensitisation Hazard Identification and Risk Assessment. Altern. Lab. Anim. 44 (5), 437–442. 10.1177/026119291604400506 27805826

[B60] Gerberick G. F. Vassallo J. D. Bailey R. E. Chaney J. G. Morrall S. W. Lepoittevin J. P. (2004). Development of a Peptide Reactivity Assay for Screening Contact Allergens. Toxicol. Sci. 81 (2), 332–343. 10.1093/toxsci/kfh213 15254333

[B61] Gerberick G. F. Vassallo J. D. Foertsch L. M. Price B. B. Chaney J. G. Lepoittevin J.-P. (2007b). Quantification of Chemical Peptide Reactivity for Screening Contact Allergens: a Classification Tree Model Approach. Toxicol. Sci. 97 (2), 417–427. 10.1093/toxsci/kfm064 17400584

[B62] Ghosh S. Kar S. Leszczynski J. (2020). “Ecotoxicity Databases for QSAR Modeling,” in Ecotoxicological QSARs. Editor Roy K. (New York, NY: Springer US), 709–758. 10.1007/978-1-0716-0150-1_29

[B63] Gilmour N. Kern P. S. Alépée N. Boislève F. Bury D. Clouet E. (2020). Development of a Next Generation Risk Assessment Framework for the Evaluation of Skin Sensitisation of Cosmetic Ingredients. Regul. Toxicol. Pharmacol. 116, 104721. 10.1016/j.yrtph.2020.104721 32645429

[B64] Gleeson D. Gleeson M. P. (2020). Theoretical Studies to Estimate the Skin Sensitization Potential of Chemicals of the Schiff Base Domain. Int. J. Quan. Chem. 120 (12), e26218. 10.1002/qua.26218

[B65] Golden E. Macmillan D. S. Dameron G. Kern P. Hartung T. Maertens A. (2021). Evaluation of the Global Performance of Eight *In Silico* Skin Sensitization Models Using Human Data. ALTEX 38 (1), 33–48. 10.14573/altex.1911261 32388570 PMC11316520

[B66] Gomolin A. Netchiporouk E. Gniadecki R. Litvinov I. V. (2020). Artificial Intelligence Applications in Dermatology: Where Do We Stand? Front. Med. 7.100 10.3389/fmed.2020.00100 PMC7136423 32296706

[B67] Graham C. Gealy R. Macina O. T. Karol M. H. Rosenkranz H. S. (1996). QSAR for Allergic Contact Dermatitis. Quant. Struct.-Act. Relat. 15, 224–229. 10.1002/qsar.19960150307 8793531

[B68] Hartung T. Blaauboer B. J. Bosgra S. Carney E. Coenen J. Conolly R. B. (2011). An Expert Consortium Review of the EC-Commissioned Report "Alternative (Non-animal) Methods for Cosmetics Testing: Current Status and Future Prospects - 2010". ALTEX 28 (3), 183–209. 10.14573/altex.2011.3.183 21993956

[B69] Hirota M. Fukui S. Okamoto K. Kurotani S. Imai N. Fujishiro M. (2015). Evaluation of Combinations Ofin Vitrosensitization Test Descriptors for the Artificial Neural Network-Based Risk Assessment Model of Skin Sensitization. J. Appl. Toxicol. 35 (11), 1333–1347. 10.1002/jat.3105 25824844

[B70] Hirota M. Kouzuki H. Ashikaga T. Sono S. Tsujita K. Sasa H. (2013). Artificial Neural Network Analysis of Data from Multiple *In Vitro* Assays for Prediction of Skin Sensitization Potency of Chemicals. Toxicol. Vitro 27 (4), 1233–1246. 10.1016/j.tiv.2013.02.013 23458967

[B71] Hirota M. Moro O. (2006). MIP-1β, a Novel Biomarker for In Vitro Sensitization Test Using Human Monocytic Cell Line. Toxicol. Vitro 20 (5), 736–742. 10.1016/j.tiv.2005.10.013 16314067

[B72] Hoffmann S. Kleinstreuer N. Alépée N. Allen D. Api A. M. Ashikaga T. (2018). Non-animal Methods to Predict Skin Sensitization (I): the Cosmetics Europe Database. Crit. Rev. Toxicol. 48 (5), 344–358. 10.1080/10408444.2018.1429385 29474128

[B73] Huppert C. Paris C. Langonné I. Muller S. Mathiot J. Abdessadeq H. (2018). Activation of T Cells by Dendritic Cells Exposed to a Reference Sensitizer: Towards a Promising Model to Assess the Allergenic Potential of Chemicals. Contact Dermatitis 79, 67–75. 10.1111/cod.12991 29635784

[B74] ICCVAM. (2011). ICCVAM Test Method Evaluation Report: Usefulness and Limitations of the Murine Local Lymph Node Assay for Potency Categorization of Chemicals Causing Allergic Contact Dermatitis in Humans.

[B75] Imamura M. Wanibuchi S. Yamamoto Y. Kojima H. Ono A. Kasahara T. (2021). Improving Predictive Capacity of the Amino Acid Derivative Reactivity Assay Test Method for Skin Sensitization Potential with an Optimal Molar Concentration of Test Chemical Solution. J. Appl. Toxicol. 41 (2), 303–329. 10.1002/jat.4082 33124715

[B76] Ivanova H. Dimitrova G. Kuseva C. Schultz T. W. Mekenyan O. G. (2020). Modeling Hazard Assessment of Chemicals Based on Adducts Formation. I. A Basis for Inclusion of Kinetic Factors in Simulating Skin Sensitization. Comput. Toxicol. 15, 100130. 10.1016/j.comtox.2020.100130

[B77] Jaworska J. Dancik Y. Kern P. Gerberick F. Natsch A. (2013). Bayesian Integrated Testing Strategy to Assess Skin Sensitization Potency: From Theory to Practice. J. Appl. Toxicol. 33 (11), a–n. 10.1002/jat.2869 23670904

[B78] Jaworska J. Dimitrov S. Nikolova N. Mekenyan O. (2002). Probabilistic Assessment of Biodegradability Based on Metabolic Pathways: Catabol System. SAR QSAR Environ. Res. 13 (2), 307–323. 10.1080/10629360290002794 12071658

[B79] Jaworska J. Harol A. Kern P. S. Gerberick G. F. (2011). Integrating Non-animal Test Information into an Adaptive Testing Strategy - Skin Sensitization Proof of Concept Case. ALTEX 28 (3), 211–225. 10.14573/altex.2011.3.211 21993957

[B80] Jaworska J. S. Natsch A. Ryan C. Strickland J. Ashikaga T. Miyazawa M. (2015). Bayesian Integrated Testing Strategy (ITS) for Skin Sensitization Potency Assessment: A Decision Support System for Quantitative Weight of Evidence and Adaptive Testing Strategy. Arch. Toxicol. 89 (12), 2355–2383. 10.1007/s00204-015-1634-2 26612363

[B81] Johansson H. Albrekt A.-S. Borrebaeck C. A. K. Lindstedt M. (2013). The GARD Assay for Assessment of Chemical Skin Sensitizers. Toxicol. Vitro 27 (3), 1163–1169. 10.1016/j.tiv.2012.05.019 23032079

[B82] Johansson H. Gradin R. Johansson A. Adriaens E. Edwards A. Zuckerstätter V. (2019). Validation of the GARDskin Assay for Assessment of Chemical Skin Sensitizers: Ring Trial Results of Predictive Performance and Reproducibility. Toxicol. Sci. 170 (2), 374–381. 10.1093/toxsci/kfz108 31099396 PMC6657565

[B83] Johansson H. Lindstedt M. Albrekt A.-S. Borrebaeck C. A. (2011). A Genomic Biomarker Signature Can Predict Skin Sensitizers Using a Cell-Based *In Vitro* Alternative to Animal Tests. BMC Genomics 12, 399. 10.1186/1471-2164-12-399 21824406 PMC3176258

[B84] Johnson C. Ahlberg E. Anger L. T. Beilke L. Benigni R. Bercu J. (2020). Skin Sensitization *In Silico* Protocol. Regul. Toxicol. Pharmacol. 116, 104688. 10.1016/j.yrtph.2020.104688 32621976 PMC7518315

[B85] Kamei Y. Sueyoshi M. Hayashi K.-i. Terada R. Nozaki H. (2009). The Novel Anti*-Propionibacterium* Acnes Compound, Sargafuran, Found in the Marine Brown Alga *Sargassum Macrocarpum* . J. Antibiot. 62 (5), 259–263. 10.1038/ja.2009.25 19329987

[B86] Karlberg A.-T. Magnusson K. Nilsson U. (1992). Air Oxidation Ofd-Limonene (The Citrus Solvent) Creates Potent Allergens. Contact Dermatitis 26 (5), 332–340. 10.1111/j.1600-0536.1992.tb00129.x 1395597

[B87] Khan K. Sanderson H. Roy K. (2020). “Ecotoxicological QSARs of Personal Care Products and Biocides,”Ecotoxicological QSARs. Editor Roy K. (New York, NY: Springer US), 357–386. 10.1007/978-1-0716-0150-1_16

[B88] Kimber I. Basketter D. A. Berthold K. Butler M. Garrigue J. L. Lea L. (2001). Skin Sensitization Testing in Potency and Risk Assessment. Toxicol. Sci. 59 (2), 198–208. 10.1093/toxsci/59.2.198 11158712

[B89] Kleinstreuer N. C. Hoffmann S. Alépée N. Allen D. Ashikaga T. Casey W. (2018). Non-animal Methods to Predict Skin Sensitization (II): an Assessment of Defined Approaches. Crit. Rev. Toxicol. 48 (5), 359–374. 10.1080/10408444.2018.1429386 29474122 PMC7393691

[B90] Kligman A. M. Epstein W. (1975). Updating the Maximization Test for Identifying Contact Allergens. Contact Dermatitis 1 (4), 231–239. 10.1111/j.1600-0536.1975.tb05389.x 1235254

[B91] Kligman A. M. (1966). The Identification of Contact Allergens by Human Assay. J. Invest. Dermatol. 47 (5), 393–409. 10.1038/jid.1966.160 5924294

[B92] Klimek-Szczykutowicz M. Szopa A. Ekiert H. (2020). Citrus Limon (Lemon) Phenomenon-A Review of the Chemistry, Pharmacological Properties, Applications in the Modern Pharmaceutical, Food, and Cosmetics Industries, and Biotechnological Studies. Plants 9, 119. 10.3390/plants9010119 PMC7020168 31963590

[B93] Klopman G. (1992). MULTICASE 1. A Hierarchical Computer Automated Structure Evaluation Program. Quant. Struct.-Act. Relat. 11, 176–184. 10.1002/qsar.19920110208

[B94] Kohtarou Y. (2010). Generating two-class classification model for predicting chemical toxicity. U.S. Patent No. US 7725413 B2.

[B95] Kostal J. Voutchkova-Kostal A. (2016). CADRE-SS, an *In Silico* Tool for Predicting Skin Sensitization Potential Based on Modeling of Molecular Interactions. Chem. Res. Toxicol. 29 (1), 58–64. 10.1021/acs.chemrestox.5b00392 26650775

[B96] Kostal J. Voutchkova-Kostal A. (2020). Going All in: A Strategic Investment in In S*ilico* Toxicology. Chem. Res. Toxicol. 33 (4), 880–888. 10.1021/acs.chemrestox.9b00497 32166946

[B97] Kraft J. N. Lynde C. W. (2005). Moisturizers: what They Are and a Practical Approach to Product Selection. Skin Ther. Lett 10 (5), 1–8. 15986082

[B98] Kumar S. (2005). Exploratory Analysis of Global Cosmetic Industry: Major Players, Technology and Market Trends. Technovation 25 (11), 1263–1272. 10.1016/j.technovation.2004.07.003

[B99] Ladizinski B. Mistry N. Kundu R. V. (2011). Widespread Use of Toxic Skin Lightening Compounds: Medical and Psychosocial Aspects. Dermatol. Clin. 29 (1), 111–123. 10.1016/j.det.2010.08.010 21095535

[B100] Langton K. Patlewicz G. Y. Long A. Marchant C. A. Basketter D. A. (2006). Structure?activity Relationships for Skin Sensitization: Recent Improvements to Derek for Windows. Contact Dermatitis 55 (6), 342–347. 10.1111/j.1600-0536.2006.00969.x 17101009

[B101] Lee A. Thomson J. (1999). “Drug-induced Skin Reactions,” in Adverse Drug Reactions (London: Pharmaceutical Press), 262, 125–156.

[B102] Lee S. Greenstein T. Shi L. Maguire T. Schloss R. Yarmush M. (2018). Tri-culture System for Pro-hapten Sensitizer Identification and Potency Classification. Technol. 06 (2), 67–74. 10.1142/s233954781850005x PMC6276108 30519598

[B103] Leontaridou M. Urbisch D. Kolle S. N. Ott K. Mulliner D. S. Gabbert S. (2017). The Borderline Range of Toxicological Methods: Quantification and Implications for Evaluating Precision. ALTEX 34 (4), 525–538. 10.14573/altex.1606271 28230889

[B104] Macmillan D. S. Chilton M. L. (2019). A Defined Approach for Predicting Skin Sensitisation Hazard and Potency Based on the Guided Integration of *In Silico, in Chemico* and *In Vitro* Data Using Exclusion Criteria. Regul. Toxicol. Pharmacol. 101, 35–47. 10.1016/j.yrtph.2018.11.001 30439387

[B105] Madden J. C. Enoch S. J. Paini A. Cronin M. T. D. (2020). A Review of *In Silico* Tools as Alternatives to Animal Testing: Principles, Resources and Applications. Altern. Lab. Anim. 48 (4), 146–172. 10.1177/0261192920965977 33119417

[B106] Mahé A. (2014). The Practice of Skin‐Bleaching for a Cosmetic Purpose in Immigrant Communities. J. Trav. Med. 21 (4), 282–287. 10.1111/jtm.12106 24612323

[B107] Martin K. I. Glaser D. A. (2011). Cosmeceuticals: the New Medicine of Beauty. Mo. Med. 108 (1), 60–63. 21462614 PMC6188460

[B108] Martin S. F. Esser P. R. Schmucker S. Dietz L. Naisbitt D. J. Park B. K. (2010). T-cell Recognition of Chemicals, Protein Allergens and Drugs: towards the Development of *In Vitro* Assays. Cell. Mol. Life Sci. 67 (24), 4171–4184. 10.1007/s00018-010-0495-3 20717835 PMC11115584

[B109] Marzulli F. N. Maibach H. I. (1974). The Use of Graded Concentrations in Studying Skin Sensitizers: Experimental Contact Sensitization in Man. Food Cosmetics Toxicol. 12 (2), 219–227. 10.1016/0015-6264(74)90367-8 4459237

[B110] Masinja W. Elliott C. Modi S. Enoch S. J. Cronin M. T. D. McInnes E. F. (2021). Comparison of the Predictive Nature of the Genomic Allergen Rapid Detection (GARD) Assay with Mammalian Assays in Determining the Skin Sensitisation Potential of Agrochemical Active Ingredients. Toxicol. Vitro 70, 105017. 10.1016/j.tiv.2020.105017 33038465

[B111] Matsumura K. (2020). Skin Sensitizer Classification Using Dual-Input Machine Learning Model. Cbij 20, 54–57. 10.1273/cbij.20.54

[B114] Mekenyan O. Dimitrov S. Pavlov T. Dimitrova G. Todorov M. Petkov P. (2012). Simulation of Chemical Metabolism for Fate and Hazard Assessment. V. Mammalian Hazard Assessment. SAR QSAR Environ. Res. 23 (5-6), 553–606. 10.1080/1062936x.2012.679689 22536822

[B115] Natsch A. Emter R. (2008). Skin Sensitizers Induce Antioxidant Response Element Dependent Genes: Application to the *In Vitro* Testing of the Sensitization Potential of Chemicals. Toxicol. Sci. 102 (1), 110–119. 10.1093/toxsci/kfm259 17932397

[B116] Natsch A. Gfeller H. (2008). LC-MS-based Characterization of the Peptide Reactivity of Chemicals to Improve the *In Vitro* Prediction of the Skin Sensitization Potential. Toxicol. Sci. 106 (2), 464–478. 10.1093/toxsci/kfn194 18791182

[B117] Natsch A. Gfeller H. Rothaupt M. Ellis G. (2007). Utility and Limitations of a Peptide Reactivity Assay to Predict Fragrance Allergens *In Vitro* . Toxicol. Vitro 21 (7), 1220–1226. 10.1016/j.tiv.2007.03.016 17513083

[B118] Natsch A. Haupt T. Wareing B. Landsiedel R. Kolle S. N. J. A.-A. t. a. e. (2020). Predictivity of the Kinetic Direct Peptide Reactivity Assay (kDPRA) for Sensitizer Potency Assessment and Subclassification. Altex 37 (4), 652–664. 10.14573/altex.2004292 32840629

[B119] Natsch A. Ryan C. A. Foertsch L. Emter R. Jaworska J. Gerberick F. (2013). A Dataset on 145 Chemicals Tested in Alternative Assays for Skin Sensitization Undergoing Prevalidation. J. Appl. Toxicol. 33, a1337–n1352. 10.1002/jat.2868 23576290

[B120] Netzlaff F. Lehr C.-M. Wertz P. W. Schaefer U. F. (2005). The Human Epidermis Models EpiSkin, SkinEthic and EpiDerm: an Evaluation of Morphology and Their Suitability for Testing Phototoxicity, Irritancy, Corrosivity, and Substance Transport. Eur. J. Pharmaceutics Biopharmaceutics 60 (2), 167–178. 10.1016/j.ejpb.2005.03.004 15913972

[B121] Nilsson A.-M. Bergström M. A. Luthman K. Nilsson J. L. G. Karlberg A.-T. (2005). A Conjugated Diene Identified as a Prohapten: Contact Allergenic Activity and Chemical Reactivity of Proposed Epoxide Metabolites. Chem. Res. Toxicol. 18 (2), 308–316. 10.1021/tx049758c 15720137

[B122] Nukada Y. Ashikaga T. Sakaguchi H. Sono S. Mugita N. Hirota M. (2011). Predictive Performance for Human Skin Sensitizing Potential of the Human Cell Line Activation Test (H-CLAT). Contact Dermatitis 65 (6), 343–353. 10.1111/j.1600-0536.2011.01952.x 21767275

[B123] Nukada Y. Miyazawa M. Kazutoshi S. Sakaguchi H. Nishiyama N. (2013). Data Integration of Non-animal Tests for the Development of a Test Battery to Predict the Skin Sensitizing Potential and Potency of Chemicals. Toxicol. Vitro 27 (2), 609–618. 10.1016/j.tiv.2012.11.006 23149339

[B124] Nuriya S. Yagita H. Okumura K. Azuma M. (1996). The Differential Role of CD86 and CD80 Co-stimulatory Molecules in the Induction and the Effector Phases of Contact Hypersensitivity. Int. Immunol. 8 (6), 917–926. 10.1093/intimm/8.6.917 8671681

[B125] Ochoa M. T. Loncaric A. Krutzik S. R. Becker T. C. Modlin R. L. (2008). "Dermal Dendritic Cells" Comprise Two Distinct Populations: CD1+ Dendritic Cells and CD209+ Macrophages. J. Invest. Dermatol. 128 (9), 2225–2231. 10.1038/jid.2008.56 18337829 PMC2682223

[B126] OECD (2016b). *In vitro* skin sensitization: U937 Cell Line Activation Test (U-SENS^TM^). Paris, France: OECD.

[B127] OECD (2018a). In vitro skin sensitisation assays addressing the AOP Key Event on Keratinocyte Activation. Paris, France: OECD.

[B128] OECD (2017). Draft key event based test guidelines 442D: In Vitro Skin Sensitisation Assays Addressing the AOP Key Event on: Keratinocyte Activation. Paris, France: OECD.

[B129] OECD (2020). Draft OECD Guideline for the Testing of Chemicals: Key-Event-Based Test Guideline for In Chemico Skin Sensitization Assays Addressing the Adverse Outcome Pathway Key Event on Covalent Binding to Proteins. Paris, France: OECD.

[B130] OECD (2016a). Guidance Document on the Reporting of Defined Approaches and Individual Information Sources to Be Used within Integrated Approaches to Testing and Assessment (IATA) for Skin Sensitisation. Paris, France: OECD.

[B131] OECD (2018b). Key Event Based Test Guideline 442Dvitro Skin Sensitization Assays Addressing the AOP Key Event on Keratinocyte Activation. Paris, France: OECD.

[B132] OECD (1992). Test No. 406OECD guideline Test. Chemicals- Skin Sensitisation. Paris, France: OECD.

[B133] OECD (2010a). Test No. 429 Skin Sensitization: Local Lymph Node Assay. Paris, France: OECD.

[B134] OECD (2019). Test No. 439: In vitro Skin Irritation: Reconstructed Human Epidermis Test. Method, . Paris, France: OECD.

[B135] OECD (2010b). Test No. 442A Skin Sensitization: Local Lymph Node Assay DA. Paris, France: OECD.

[B136] OECD (2015a). Test No. 442C: In Chemico Skin Sensitisation: Direct Peptide Reactivity Assay (DPRA). Paris, France: OECD.

[B137] OECD (2015b). Test No. 442D: In Vitro Skin Sensitisation: ARE-Nrf2 luciferase test method. Paris, France: OECD.

[B138] OECD (2018c). Test No. 442E: In Vitro Skin Sensitisation Assays Addressing the Key Event on Activation of Dendritic Cells on the Adverse Outcome Pathway for Skin Sensitisation. Paris, France: OECD.

[B139] OECD (2012). The Adverse Outcome Pathway for Skin Sensitisation Initiated by Covalent Binding to Proteins, Paris, France: OECD.

[B140] Ohtake T. Maeda Y. Hayashi T. Yamanaka H. Nakai M. Takeyoshi M. (2018). Applicability of an Integrated Testing Strategy Consisting of In Silico , in Chemico and In Vitro Assays for Evaluating the Skin Sensitization Potencies of Isocyanates. Toxicology 393, 9–14. 10.1016/j.tox.2017.10.015 29100879

[B141] Olumide Y. M. Akinkugbe A. O. Altraide D. Mohammed T. Ahamefule N. Ayanlowo S. (2008). Complications of Chronic Use of Skin Lightening Cosmetics. Int. J. Dermatol. 47 (4), 344–353. 10.1111/j.1365-4632.2008.02719.x 18377596

[B142] Orbis-Research (2018).Global Cosmetics Products Market-Analysis of Growth Trends Forecasts. Mordor Intelligence.

[B143] Otsubo Y. Nishijo T. Miyazawa M. Saito K. Mizumachi H. Sakaguchi H. (2017). Binary Test Battery with KeratinoSens and H-CLAT as Part of a Bottom-Up Approach for Skin Sensitization Hazard Prediction. Regul. Toxicol. Pharmacol. 88, 118–124. 10.1016/j.yrtph.2017.06.002 28602621

[B144] Ouyang Q. Wang L. Mu Y. Xie X.-Q. (2014). Modeling Skin Sensitization Potential of Mechanistically Hard-To-Be-Classified Aniline and Phenol Compounds with Quantum Mechanistic Properties. BMC Pharmacol. Toxicol. 15, 76. 10.1186/2050-6511-15-76 25539579 PMC4298069

[B145] Pandey A. Jatana G. K. Sonthalia S. (2021). Cosmeceuticals. In StatPearls. Treasure Island (FL): StatPearls Publishing Copyright © 2021, StatPearls Publishing LLC.

[B146] Panico A. Serio F. Bagordo F. Grassi T. Idolo A. De Giorgi M. (2019). Skin Safety and Health Prevention: an Overview of Chemicals in Cosmetic Products. J. Prev. Med. Hyg. 60 (1), E50–E57. 10.15167/2421-4248/jpmh2019.60.1.1080 31041411 PMC6477564

[B147] Piroird C. Ovigne J.-M. Rousset F. Martinozzi-Teissier S. Gomes C. Cotovio J. (2015). The Myeloid U937 Skin Sensitization Test (U-SENS) Addresses the Activation of Dendritic Cell Event in the Adverse Outcome Pathway for Skin Sensitization. Toxicol. Vitro 29 (5), 901–916. 10.1016/j.tiv.2015.03.009 25820135

[B148] Posadas S. J. Pichler W. J. (2007). Delayed Drug Hypersensitivity Reactions ? New Concepts. Clin. Exp. Allergy 37 (7), 989–999. 10.1111/j.1365-2222.2007.02742.x 17581192

[B149] Python F. Goebel C. Aeby P. (2007). Assessment of the U937 Cell Line for the Detection of Contact Allergens. Toxicol. Appl. Pharmacol. 220 (2), 113–124. 10.1016/j.taap.2006.12.026 17306317

[B150] Ramirez T. Mehling A. Kolle S. N. Wruck C. J. Teubner W. Eltze T. (2014). LuSens: A Keratinocyte Based ARE Reporter Gene Assay for Use in Integrated Testing Strategies for Skin Sensitization Hazard Identification. Toxicol. Vitro 28 (8), 1482–1497. 10.1016/j.tiv.2014.08.002 25172300

[B151] Rehfeld A. Nylander M. Karnov K. (2017). The Integumentary System. In Compendium of Histology. Springer Nature, 411–432. 10.1007/978-3-319-41873-5_20

[B152] Reiser H. Schneeberger E. E. (1996). Expression and Function of B7-1 and B7-2 in Hapten-Induced Contact Sensitivity. Eur. J. Immunol. 26 (4), 880–885. 10.1002/eji.1830260424 8625983

[B153] Roberts D. W. Api A. M. (2018). Chemical Applicability Domain of the Local Lymph Node Assay (LLNA) for Skin Sensitisation Potency. Part 4. Quantitative Correlation of LLNA Potency with Human Potency. Regul. Toxicol. Pharmacol. 96, 76–84. 10.1016/j.yrtph.2018.04.022 29730445

[B154] Roberts D. W. (2018). Is a Combination of Assays Really Needed for Non-animal Prediction of Skin Sensitization Potential? Performance of the GARD (Genomic Allergen Rapid Detection) Assay in Comparison with OECD Guideline Assays Alone and in Combination. Regul. Toxicol. Pharmacol. 98, 155–160. 10.1016/j.yrtph.2018.07.014 30048705

[B155] Roberts D. W. Lepoittevin J. P. (1998). Hapten-protein Interactions. Berlin, Heidelberg: Springer. 81–111. 10.1007/978-3-642-80331-4_6

[B156] Roberts D. W. Patlewicz G. (2018). Non-animal Assessment of Skin Sensitization Hazard: Is an Integrated Testing Strategy Needed, and if So what Should Be Integrated? J. Appl. Toxicol. 38 (1), 41–50. 10.1002/jat.3479 28543848

[B157] Robinson M. K. Gerberick G. F. Ryan C. A. McNamee P. White I. R. Basketter D. A. (2000). The Importance of Exposure Estimation in the Assessment of Skin Sensitization Risk. Contact Dermatitis 42 (5), 251–259. 10.1034/j.1600-0536.2000.042005251.x 10789838

[B158] Rollin B. E. (2003). Toxicology and New Social Ethics for Animals. Toxicol. Pathol. 31 (Suppl. l), 128–131. 10.1080/01926230390175011 12597441

[B159] Roman D. L. Isvoran A. Filip M. Ostafe V. Zinn M. (2020). *In Silico* Assessment of Pharmacological Profile of Low Molecular Weight Oligo-Hydroxyalkanoates. Front. Bioeng. Biotechnol. 8 (1352). 10.3389/fbioe.2020.584010 PMC7726197 33324621

[B160] Saiakhov R. Chakravarti S. Klopman G. (2013). Effectiveness of CASE Ultra Expert System in Evaluating Adverse Effects of Drugs. Mol. Inf. 32 (1), 87–97. 10.1002/minf.201200081 27481026

[B161] Sakaguchi H. Ashikaga T. Miyazawa M. Kosaka N. Ito Y. Yoneyama K. (2009). The Relationship between CD86/CD54 Expression and THP-1 Cell Viability in an In Vitro Skin Sensitization Test - Human Cell Line Activation Test (H-CLAT). Cell Biol. Toxicol. 25 (2), 109–126. 10.1007/s10565-008-9059-9 18204907

[B162] Sakaguchi H. Ryan C. Ovigne J.-M. Schroeder K. R. Ashikaga T. (2010). Predicting Skin Sensitization Potential and Inter-laboratory Reproducibility of a Human Cell Line Activation Test (H-CLAT) in the European Cosmetics Association (COLIPA) Ring Trials. Toxicol. Vitro 24 (6), 1810–1820. 10.1016/j.tiv.2010.05.012 20510347

[B163] Sakaguchi H. Ashikaga T. Ashikaga T. Yoshida Y. Ito Y. Yoneyama K. (2006). Development of an In Vitro Skin Sensitization Test Using Human Cell Lines; Human Cell Line Activation Test (H-CLAT) II. An Inter-laboratory Study of the H-CLAT. Toxicol. Vitro 20 (5), 774–784. 10.1016/j.tiv.2005.10.014 16337770

[B164] Sato K. Umemura T. Tamura T. Kusaka Y. Aoyama K. Ueda A. (2012). Skin Sensitization Study by a New Qualitative Structure-Toxicity Relationships (QSTR) Approach: K-step Yard Sampling (KY) Methods. J. Oral Tissue Engin. 9 (3), 167–173. 10.11223/jarde.9.167

[B165] Sato K. Umemura T. Tamura T. Kusaka Y. Aoyama K. Ueda A. (2009). Skin Sensitization Study by Quantitative Structure-Activity Relationships (QSAR). Alternatives Anim. Test. Experimentation 14 (3), 940–946. 10.11232/aatex.14.940

[B166] Schweiger E. S. Weinberg J. M. (2004). Novel Antibacterial Agents for Skin and Skin Structure Infections. J. Am. Acad. Dermatol. 50 (3), 331–340. 10.1016/j.jaad.2003.10.665 14988672

[B167] Schwöbel J. A. H. Koleva Y. K. Enoch S. J. Bajot F. Hewitt M. Madden J. C. (2011). Measurement and Estimation of Electrophilic Reactivity for Predictive Toxicology. Chem. Rev. 111 (4), 2562–2596. 10.1021/cr100098n 21401043

[B168] Serio F. Pizzolante G. Cozzolino G. D’Alba M. Bagordo F. De Giorgi M. (2017). A New Formulation Based on Ozonated Sunflower Seed Oil: *In vitro* Antibacterial and Safety Evaluation. Ozone: Sci. Eng. 39 (3), 139–147. 10.1080/01919512.2016.1272405

[B169] Silva F. A. L. S. Brites G. Ferreira I. Silva A. Miguel Neves B. Costa Pereira J. L. G. F. S. (2020). Evaluating Skin Sensitization via Soft and Hard Multivariate Modeling. Int. J. Toxicol. 39 (6), 547–559. 10.1177/1091581820944395 32757797

[B170] Sköld M. Börje A. Matura M. Karlberg A.-T. (2002). Studies on the Autoxidation and Sensitizing Capacity of the Fragrance Chemical Linalool, Identifying a Linalool Hydroperoxide. Contact Dermatitis 46 (5), 267–272. 10.1034/j.1600-0536.2002.460504.x 12084079

[B171] Spada F. Barnes T. M. Greive K. A. (2018). Skin Hydration Is Significantly Increased by a Cream Formulated to Mimic the Skin’s Own Natural Moisturizing Systems. Ccid 11, 491–497. 10.2147/CCID.S177697 30410378 PMC6197824

[B172] Steinberg P. (2013). “High-throughput Screening Methods,”.in Toxicity Testing Editor Steinberg P. 1st ed. (Hoboken, NJ: John Wiley & Sons).

[B173] Stevenson M. Czekala L. Simms L. Tschierske N. Larne O. Walele T. (2019). The Use of Genomic Allergen Rapid Detection (GARD) Assays to Predict the Respiratory and Skin Sensitising Potential of E-Liquids. Regul. Toxicol. Pharmacol. 103, 158–165. 10.1016/j.yrtph.2019.01.001 30629970

[B174] Strickland J. Daniel A. B. Allen D. Aguila C. Ahir S. Bancos S. (2019). Skin Sensitization Testing Needs and Data Uses by US Regulatory and Research Agencies. Arch. Toxicol. 93 (2), 273–291. 10.1007/s00204-018-2341-6 30377734 PMC6363849

[B175] Strickland J. Zang Q. Kleinstreuer N. Paris M. Lehmann D. M. Choksi N. (2016). Integrated Decision Strategies for Skin Sensitization Hazard. J. Appl. Toxicol. 36 (9), 1150–1162. 10.1002/jat.3281 26851134 PMC4945438

[B176] Strickland J. Zang Q. Paris M. Lehmann D. M. Allen D. Choksi N. (2017). Multivariate Models for Prediction of Human Skin Sensitization Hazard. J. Appl. Toxicol. 37 (3), 347–360. 10.1002/jat.3366 27480324 PMC5243794

[B177] Sushko I. Novotarskyi S. Körner R. Pandey A. K. Rupp M. Teetz W. (2011). Online Chemical Modeling Environment (OCHEM): Web Platform for Data Storage, Model Development and Publishing of Chemical Information. J. Comput. Aided Mol. Des. 25 (6), 533–554. 10.1007/s10822-011-9440-2 21660515 PMC3131510

[B178] Sushko I. Salmina E. Potemkin V. A. Poda G. Tetko I. V. (2012). ToxAlerts: A Web Server of Structural Alerts for Toxic Chemicals and Compounds with Potential Adverse Reactions. J. Chem. Inf. Model. 52 (8), 2310–2316. 10.1021/ci300245q 22876798 PMC3640409

[B179] Suzuki M. Hirota M. Hagino S. Itagaki H. Aiba S. (2009). Evaluation of Changes of Cell-Surface Thiols as a New Biomarker for *In Vitro* Sensitization Test. Toxicol. Vitro 23 (4), 687–696. 10.1016/j.tiv.2009.02.002 19490838

[B180] Takenouchi O. Fukui S. Okamoto K. Kurotani S. Imai N. Fujishiro M. (2015). Test Battery with the Human Cell Line Activation Test, Direct Peptide Reactivity Assay and DEREK Based on a 139 Chemical Data Set for Predicting Skin Sensitizing Potential and Potency of Chemicals. J. Appl. Toxicol. 35 (11), 1318–1332. 10.1002/jat.3127 25820183

[B181] Takenouchi O. Miyazawa M. Saito K. Ashikaga T. Sakaguchi H. (2013). Predictive Performance of the Human Cell Line Activation Test (H-CLAT) for Lipophilic Chemicals with High Octanol-Water Partition Coefficients. J. Toxicol. Sci. 38 (4), 599–609. 10.2131/jts.38.599 23824015

[B182] Tarca A. L. Carey V. J. Chen X.-w. Romero R. Drăghici S. (2007). Machine Learning and its Applications to Biology. Plos Comput. Biol. 3, e116. 10.1371/journal.pcbi.0030116 17604446 PMC1904382

[B183] Teubner W. Mehling A. Schuster P. X. Guth K. Worth A. Burton J. (2013). Computer Models *versus* Reality: How Well Do *In Silico* Models Currently Predict the Sensitization Potential of a Substance. Regul. Toxicol. Pharmacol. 67 (3), 468–485. 10.1016/j.yrtph.2013.09.007 24090701

[B184] Thyssen J. P. Linneberg A. Menné T. Johansen J. D. (2007). The Epidemiology of Contact Allergy in the General Population - Prevalence and Main Findings. Contact Dermatitis 57 (5), 287–299. 10.1111/j.1600-0536.2007.01220.x 17937743

[B185] Toropov A. A. Toropova A. P. Benfenati E. Gini G. Leszczynska D. Leszczynski J. (2013). CORAL: QSPR Model of Water Solubility Based on Local and Global SMILES Attributes. Chemosphere 90 (2), 877–880. 10.1016/j.chemosphere.2012.07.035 22921649

[B186] Toropova A. P. Toropov A. A. (2017). Hybrid Optimal Descriptors as a Tool to Predict Skin Sensitization in Accordance to OECD Principles. Toxicol. Lett. 275, 57–66. 10.1016/j.toxlet.2017.03.023 28359801

[B187] Tourneix F. Alépée N. Detroyer A. Eilstein J. Ez-Zoubir M. Teissier S. M. (2020). Skin Sensitisation Testing in Practice: Applying a Stacking Meta Model to Cosmetic Ingredients. Toxicol. Vitro 66, 104831. 10.1016/j.tiv.2020.104831 32198056

[B188] Tsujita-Inoue K. Hirota M. Ashikaga T. Atobe T. Kouzuki H. Aiba S. (2014). Skin Sensitization Risk Assessment Model Using Artificial Neural Network Analysis of Data from Multiple *In Vitro* Assays. Toxicol. Vitro 28 (4), 626–639. 10.1016/j.tiv.2014.01.003 24444449

[B189] Tung C.-W. Lin Y.-H. Wang S.-S. (2019). Transfer Learning for Predicting Human Skin Sensitizers. Arch. Toxicol. 93 (4), 931–940. 10.1007/s00204-019-02420-x 30806762

[B190] Tung C.-W. Wang C.-C. Wang S.-S. (2018). Mechanism-informed Read-Across Assessment of Skin Sensitizers Based on SkinSensDB. Regul. Toxicol. Pharmacol. 94, 276–282. 10.1016/j.yrtph.2018.02.014 29486270

[B191] Tuschl H. Kovac R. Weber E. (2000). The Expression of Surface Markers on Dendritic Cells as Indicators for the Sensitizing Potential of Chemicals. Toxicol. Vitro 14 (6), 541–549. 10.1016/s0887-2333(00)00051-5 11033066

[B192] United Nation. (2013). Globally Harmonized System of Classification and Labelling of Chemicals (GHS).

[B193] Urbisch D. Honarvar N. Kolle S. N. Mehling A. Ramirez T. Teubner W. (2016). Peptide Reactivity Associated with Skin Sensitization: The QSAR Toolbox and TIMES Compared to the DPRA. Toxicol. Vitro 34, 194–203. 10.1016/j.tiv.2016.04.005 27090964

[B194] Urbisch D. Mehling A. Guth K. Ramirez T. Honarvar N. Kolle S. (2015). Assessing Skin Sensitization Hazard in Mice and Men Using Non-animal Test Methods. Regul. Toxicol. Pharmacol. 71 (2), 337–351. 10.1016/j.yrtph.2014.12.008 25541156

[B195] van Eijl S. Zhu Z. Cupitt J. Gierula M. Götz C. Fritsche E. (2012). Elucidation of Xenobiotic Metabolism Pathways in Human Skin and Human Skin Models by Proteomic Profiling. PLoS One 7 (7), e41721. 10.1371/journal.pone.0041721 22848577 PMC3406074

[B196] Wang C.-C. Lin Y.-C. Wang S.-S. Shih C. Lin Y.-H. Tung C.-W. (2017). SkinSensDB: A Curated Database for Skin Sensitization Assays. J. Cheminform. 9, 5. 10.1186/s13321-017-0194-2 28194231 PMC5285290

[B197] Wanibuchi S. Yamamoto Y. Sato A. Kasahara T. Fujita M. (2019). The Amino Acid Derivative Reactivity Assay with Fluorescence Detection and its Application to Multi-Constituent Substances. J. Toxicol. Sci. 44 (12), 821–832. 10.2131/jts.44.821 31813901

[B198] Wareing B. Kolle S. N. Birk B. Alépée N. Haupt T. Kathawala R. (2020). The Kinetic Direct Peptide Reactivity Assay (kDPRA): Intra- and Inter-laboratory Reproducibility in a Seven-Laboratory Ring Trial. ALTEX 37 (4), 639–651. 10.14573/altex.2004291 32521036

[B199] Weng C.-F. Leong M. K. (2020). Chapter Two - In Silico Prediction of Drug-Induced Liver Injury: Quo Vadis? Elsevier.

[B200] Wilm A. Kühnl J. Kirchmair J. (2018). Computational Approaches for Skin Sensitization Prediction. Crit. Rev. Toxicol. 48 (9), 738–760. 10.1080/10408444.2018.1528207 30488745

[B201] Wilm A. Norinder U. Agea M. I. de Bruyn Kops C. Stork C. Kühnl J. (2020). Skin Doctor CP: Conformal Prediction of the Skin Sensitization Potential of Small Organic Molecules. Chem. Res. Toxicol. 34, 330–344. 10.1021/acs.chemrestox.0c00253 33295759 PMC7887802

[B202] Zang Q. Paris M. Lehmann D. M. Bell S. Kleinstreuer N. Allen D. (2017). Prediction of Skin Sensitization Potency Using Machine Learning Approaches. J. Appl. Toxicol. 37 (7), 792–805. 10.1002/jat.3424 28074598 PMC5435511

